# A Differential Metabarcoding Approach to Describe Taxonomy Profiles of *Bacteria* and *Archaea* in the Saltern of Margherita di Savoia (Italy)

**DOI:** 10.3390/microorganisms8060936

**Published:** 2020-06-22

**Authors:** Claudia Leoni, Mariateresa Volpicella, Bruno Fosso, Caterina Manzari, Elisabetta Piancone, Maria C. G. Dileo, Erika Arcadi, Michail Yakimov, Graziano Pesole, Luigi R. Ceci

**Affiliations:** 1 Institute of Biomembranes, Bioenergetics and Molecular Biotechnologies, CNR, Via Amendola 122/O, 70126 Bari, Italy; c.leoni@ibiom.cnr.it (C.L.); b.fosso@ibiom.cnr.it (B.F.); c.manzari@ibiom.cnr.it (C.M.); graziano.pesole@uniba.it (G.P.); 2 Department of Biosciences, Biotechnologies and Biopharmaceutics, University of Bari “Aldo Moro”, Via Orabona 4, 70126 Bari, Italy; e.piancone@ibiom.cnr.it (E.P.); mariacristina.g.1993@gmail.com (M.C.G.D.); 3 Stazione Zoologica Anton Dohrn, Via dei Mille 46, 98057 Milazzo (Messina), Italy; erika.arcadi@szn.it; 4 Institute for Biological Resources and Marine Biotechnology, CNR, Spianata San Raineri 86, 98122 Messina, Italy; mikhail.iakimov@cnr.it

**Keywords:** halophiles, extremophiles, microbiota, saltern

## Abstract

Microorganisms inhabiting saline environments are an interesting ecological model for the study of the adaptation of organisms to extreme living conditions and constitute a precious resource of enzymes and bioproducts for biotechnological applications. We analyzed the microbial communities in nine ponds with increasing salt concentrations (salinity range 4.9–36.0%) of the Saltern of Margherita di Savoia (Italy), the largest thalassohaline saltern in Europe. A deep-metabarcoding NGS procedure addressing separately the V5-V6 and V3-V4 hypervariable regions of the 16S rRNA gene of *Bacteria* and *Archaea*, respectively, and a CARD-FISH (catalyzed reporter deposition fluorescence in situ hybridization) analysis allowed us to profile the dynamics of microbial populations at the different salt concentrations. Both the domains were detected throughout the saltern, even if the low relative abundance of *Archaea* in the three ponds with the lowest salinities prevented the construction of the relative amplicon libraries. The highest cell counts were recorded at 14.5% salinity for *Bacteria* and at 24.1% salinity for *Archaea*. While *Bacteria* showed the greatest number of genera in the first ponds (salinity range 4.9–14.5%), archaeal genera were more numerous in the last ponds of the saltern (salinity 24.1–36.0%). Among prokaryotes, *Salinibacter* was the genus with the maximum abundance (~49% at 34.6% salinity). Other genera detected at high abundance were the archaeal *Haloquadratum* (~43% at 36.0% salinity) and *Natronomonas* (~18% at 13.1% salinity) and the bacterial “*Candidatus* Aquiluna” (~19% at 14.5% salinity). Interestingly, “*Candidatus* Aquiluna” had not been identified before in thalassohaline waters.

## 1. Introduction

Marine solar salterns are excellent systems to study the influence of salinity on microbial populations. They consist of a series of connected ponds containing water with increasing saline concentrations. The first ponds, from those directly connected with the sea up to those containing water with a saline concentration around 20%, are generally indicated as evaporation ponds. Ponds at higher salt concentrations—up to the NaCl saturation point—are known as crystallizer ponds. As salinity increases, changes in the microbial halophiles’ population occur. Generally, microbial halophiles are classified as “slight halophiles”, which grow optimally in media with low salinity values (1–5%); “moderate halophiles”, which require salt concentrations from 3 to 25% and “extreme halophiles” which grow best in media containing salt concentrations in the 20−30% range [1,2]. Both *Bacteria* and *Archaea* have been identified among halophiles [3,4,5,6,7].

Studies aimed at the identification of prokaryotes living in the waters with different salt concentrations of salterns have been carried out for a long time by using different approaches, including classic culturing methods and advanced molecular techniques [8,9,10]. Even if some general trends are evident, such as the decrease in bacterial diversity with the increase in salinity or the relative increase in archaeal diversity at high salt concentrations [6], a clear view of the dynamicity of prokaryotic taxonomic profiles in relation to the increase in salinity in the different ponds is difficult to obtain. Indeed, some noteworthy differences have been reported. For example, the haloarchaeal genus *Haloquadratum*, which is considered to be present worldwide [6,11], was not detected in the crystallizer ponds of two salterns of the Adriatic sea (Secovlje in Slovenia and Ston in Croatia). These salterns are instead particularly rich in members of the haloarchaeal *Halorubrum* genus (66 and 87% of 16S rDNA gene amplicons, respectively) [12]. The absence of the *Haloquadratum* genus was also reported in the salterns located in San Diego (California), where the *Halobacterium* prevails [13]. A recent study regarding the archaeal population in a crystallizer pond from the salterns of Pomorie (Bulgary) also highlighted the very scarcity of the genus *Haloquadratum*, and the prevailing of the genera *Halanaeroarchaeum*, *Halorubrum*, *Halonotius*, *Halobellus* and *Halovenus*, plus other less abundant archaeal genera [14]. As for *Bacteria*, in crystallizer ponds, they can be completely absent or in some cases, they reach relatively high levels of abundance (25–27%) largely due to the genus *Salinibacter* [15]. Specific geographic and environmental conditions can be the basis of these results [16]. Therefore, further studies on the microbial composition in hypersaline environments are required to get a more detailed representation of their distribution, their adaptability and their evolution. From an applicative point of view, marine salterns are also a valuable resource for the exploitation of extremophiles living at high saline concentrations, since they can be the source of unique enzymes with potential biotechnological applications [17,18,19,20,21].

In this work, we studied by a deep metabarcoding approach the prokaryotic community of the saltern of Margherita di Savoia (MdS), located on the Adriatic Sea, Apulian coast (Italy). It is the largest and most important hypersaline habitat for the production of salt in Europe and represents a still unexplored site of microbial biodiversity. In particular, the waters from nine ponds at increasing salt concentrations were studied by using two distinct metabarcoding experiments, addressing respectively the bacterial and archaeal populations. DAPI and CARD-FISH (catalyzed reporter deposition fluorescence in situ hybridization) cell count data from the same waters also contributed to obtain the overall representation of the microbial distribution at different salinities. The presence in wide ranges of salinities of specific lineages, such as *Natronomonas* and “*Candidatus* Aquiluna”, is an unexpected finding that deserves additional investigations.

## 2. Materials and Methods

### 2.1. Sampling

Water was collected from nine different ponds with increasing salinities of the MdS saltern. Sampling was carried out on 22 June 2017, during the activity period of the saltern (April–September). All the samples were constituted by the water collected into one-liter bottles, filled by horizontal circular movement of the operator’s arm at about 30 cm below the water surface of each pond. Water temperature was measured directly at the sampling site; water salinity and pH were measured in the laboratories of the MdS saltern using a conventional refractometer and pH meter (Table 1).

### 2.2. CARD-FISH Analyses

The abundance and the distribution of archaeal and bacterial populations inhabiting the MdS saltern ponds were analyzed by CARD-FISH [22]. In total, 10 mL of water samples from the nine ponds with increasing salinities were fixed for 1 h at room temperature with 2% vol/vol pre-filtered formaldehyde and filtered through 0.22 µm (Ø25 mm) polycarbonate membranes (Sartorius, Gottingen, Germany). Cells were further permeabilized by incubation for 1 h with lysozyme (10 mg/mL in TE buffer, pH 8.0) followed by incubation with achromo-peptidase (5 mg/mL) for 30 min. Both steps were performed at 37 °C. Intracellular peroxidase was inhibited by treatment with 10 mM HCl at room temperature for 20 min. Following this, the filters were washed with 0.22 µm filtered MilliQ water, dipped in 95% ethanol and air-dried. Filters were further cut into sections and cells were hybridized with universal horseradish peroxidase (HRP)-labeled oligonucleotide probes for *Bacteria* (EUB338 I, II, III probe mix) [23,24]. The presence and quantity of archaeal cells were monitored with an (HRP)-labeled oligonucleotide probe for *Archaea* (Arch915) [25] (see Table S1 in Supplementary Material). For signal amplification, tyramide-Alexa488 and tyramide-Alexa594 were used [26]. The filter sections were counter-stained with 2 μg mL^−1^ of 4′,6-diamidino-2-phenylindole (DAPI) in a four-to-one ratio of Citifluor (Citifluor Ltd., Leicester, UK) and Vectashield (Linaris GmbH, Wertheim-Bettingen, Germany). At least 200 DAPI-stained and Alexa-positive cells were counted in a minimum of 10 fields under an AXIOPLAN 2 Imaging microscope (Zeiss, Jena, Germany).

### 2.3. DNA Extraction

In total, 30 mL of water samples were pre-filtered with a system that constituted a 50 mL syringe connected to 5 µm pore size polyethersulfone membrane filters (Stericup, Merck-Millipore, Burlington, MA, USA). Pre-filtered water samples were subsequently filtered through 0.22 µm pore size polyethersulfone membrane filters. Three distinct filters were produced for each pond. Filters were stored at −20 °C until DNA extraction. Total DNA was extracted from filters by using the FastDNA SPIN Kit for Soil (MP Biomedicals, CA, USA), according to the manufacturer’s instructions. A 40 sec bead-beating step at speed 6 was applied using the FastPrep Instrument (BIO 101, Carlsbad, Canada). DNA was eluted in 100 μL and stored at −20 °C until further analysis. The quality and concentration of the DNA extracts were determined by 1% agarose gel electrophoretic analysis and by spectrophotometric measurements at 260, 280, and 230 nm using a NanoDrop^®^ ND-1000 Spectrophotometer (Thermo Fisher Scientific Inc., MI, Italy), according to consolidated procedures [27]. DNA samples were stored at −20 °C until further analyses.

### 2.4. Amplicon Library Preparation and Illumina-Based Sequencing

Amplicon libraries for *Bacteria* and *Archaea* were prepared and sequenced separately. For *Bacteria,* the V5-V6 hypervariable region of the 16S rRNA gene was amplified using the primers B-V5 and A-V6 [28,29] (Table S1).

For *Archaea,* the V3-V4 hypervariable regions of the 16S rRNA gene was amplified using the primers Arch_349F and Arch_806R [30] (Table S1).

Amounts of 5 and 10 ng of DNA extracted from each water sample were used for the preparation of the *Bacteria* and *Archaea* amplicon libraries, respectively. The strategy used to prepare the bacterial libraries was as already described in detail for the analysis of other microbiomes [31,32]. For the preparation of the archaeal libraries, the annealing temperature of the first amplification was set to 54 °C. Purified amplicons were pooled in equimolar ratio and subjected to a 2 × 250 bp paired-end sequencing on the Illumina MiSeq platform. To increase genetic diversity, as required by the MiSeq platform, 30% of phage PhiX genomic DNA library was added to the mix and co-sequenced.

### 2.5. Bioinformatic Analysis

The obtained Illumina MiSeq reads were analyzed by using a bioinformatic procedure including two main steps: denoising and taxonomical classification. The first one relies on the inference of the Amplicon Sequence Variants (ASVs, i.e., an estimation of the actual amplicons) and the latter to taxonomically annotate the inferred ASVs. In particular, raw Paired End (PE) reads were treated with Cutadapt [33] to remove Illumina adaptors and PCR primers. Following this, the resulting trimmed reads were denoised by applying the DADA2 (Divisive Amplicon Denoising Algorithm) workflow [34]. The procedure also removed chimera and PhiX sequences. The obtained ASVs were taxonomically annotated using the QIIME2 (Quantitative Insights Into Microbial Ecology) [35] plugin fit-classifier-sklearn [36] by using the release 138 of the SILVA database [37] as the 16S rRNA reference collection and taxonomy.

A phylogenetic tree was inferred by using the QIIME2 align-to-tree-mafft-fasttree plugin: a multiple sequence alignment of ASVs sequences was obtained by using MAFFT (Multiple sequence Alignment based on Fast Fourier Transform [38] and the phylogenetic tree was inferred by applying the maximum-likelihood procedure implemented in Fasttree 2 [39]. The ASVs table was normalized by using rarefaction [40] for diversity analysis.

The Shannon index (α diversity) was inferred on rarefied ASVs for all samples, including both biological and technical replicates, by applying the R-package phyloseq [41] and statistically relevant differences between the inferred measures were evaluated by using the Kruskal–Wallis test followed by the Dunn Test for pairwise comparisons.

The principal coordinates analysis (PCoA) describing the diversity between the samples (i.e., ß-diversity) based on the weighted and unweighted UNIFRAC [42] metrics, were inferred by using the ape-R package [43] and evaluated by using PERMANOVA (Permutational Multivariate Analysis Of Variance) with the vegan-R package [44].

Correlation between rarefied ASVs and salinity was measured by using Pearson’s product moment correlation coefficient. In particular, for each ASV, the mean value has been considered per pond.

Graphical representations of the inferred taxonomy were obtained by GraPhlAn (Graphical Phylogenetic Analysis) [45].

## 3. Results and Discussion

### 3.1. DAPI and CARD-FISH Analyses

**Figure 1:**
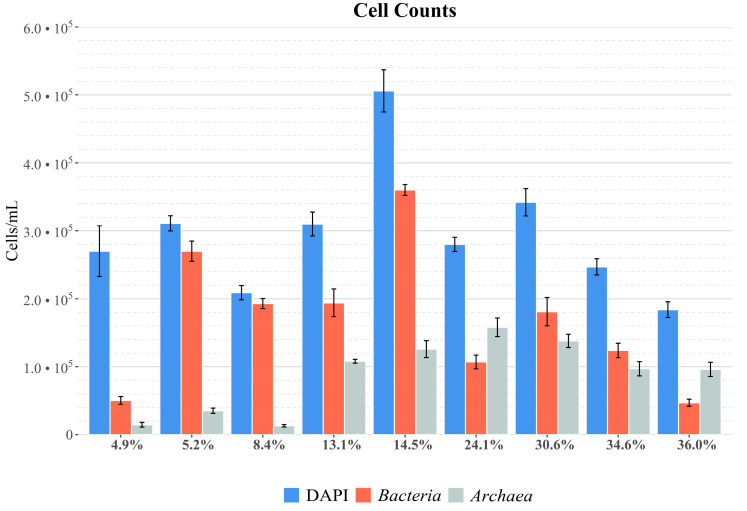
DAPI and CARD-FISH (catalyzed reporter deposition fluorescence in situ hybridization) analyses. Cell count values and relative abundances are reported in Table S2. Salinity is reported as salt percentage (w/v).

Overall microbial abundance was measured by DAPI staining, while the presence of metabolically active (ribosome-containing) cells was quantitatively assessed by CARD-FISH using *Bacteria* and *Archaea* specific probes (Figure 1). Interestingly, CARD-FISH analysis revealed the presence of *Archaea* throughout the salinity range, even if with a low abundance in the first three ponds. This well correlates with the reported impossibility to produce pure archaea 16S rRNA gene amplicons from the DNA isolated from these ponds. Relevant also was the low presence of living bacterial cells in the Alma Dannata pond (4.9% salinity). This was probably the effect on the bacterial community of the osmotic change following the passage from seawater (3.9% salinity). CARD-FISH pictures showing archaeal *Haloquadratum* and bacterial *Salinibacter* cells in the Imperatrice pond (36.0% salinity) are shown in Figure S1.

As a general trend, with the exception of the first pond, the DAPI and CARD-FISH analyses indicate a possible association between microbiota composition and salinity. In particular, in the low salinity range (up to 8.4%), *Bacteria* prevailed on *Archaea* (with an average ratio of about 10:1). The average ratio was about 2:1 in the medium salinity range (13.1–14.5%) and 1:1 in the ponds with the highest salinity (24.1–36.0%). Only in the last pond at 36.0% salinity, archaeal cells exceed twice the bacterial cells.

### 3.2. Preparation and Sequencing of 16S rRNA Gene Metabarcoding Libraries

To analyze the prokaryotic population of the ponds, we performed a deep metabarcoding analysis addressing the 16S rRNA gene and using specific pairs of primers targeting the V5-V6 hypervariable regions for *Bacteria* [28,32] and the V3-V4 hypervariable regions for *Archaea* [30]. DNA was purified from microbes collected on 0.22 micron filters after filtration of 30 mL of water from the different ponds and subjected to PCR amplifications (not shown). Three distinct samples were used for each pond. Bacterial amplicons were obtained using 5 ng of DNA. Archaeal amplicons could not be obtained from the DNA purified from the waters at the lowest saline concentrations (4.9, 5.2 and 8.4%) using up to 10 ng of DNA. Using higher amounts of DNA (20 ng), amplicons were not yet obtained from the DNA of the first two ponds and non-specific amplicons were produced from the 8.4% salinity pond. Therefore, archaeal amplicon libraries were prepared only from the six ponds with salinity from 13.1 to 36.0%, using 10 ng of DNA.

Amplicon libraries from each pond were sequenced by a 2 × 250 bp paired-end approach on an Illumina MiSeq platform. Bacterial amplicons produced about 475,287 PE reads in average per sample (SD 103,916). About 98% of them passed the trimming of adaptors and PCR primers step. For archaeal amplicons, about 168,804 PE reads were obtained on average per sample (SD 32,909), with 93% of them passing the trimming of adaptors and PCR primers step. The forty-five distinct sequencing raw data (corresponding to nine ponds in triplicate for bacterial amplicons and six pond in triplicate for archaeal amplicons) are available in the SRA (Short Read Archive) repository (PRJNA408245).

The denoising step allowed inferring of the ASVs (amplicon sequence variant) and their absolute counts. The subsequent removal of chimera and contaminant sequences retained about 62% of the bacterial ASVs and about 66% of archaeal ASVs. ASVs were finally filtered to remove very low abundant ASVs (relative abundance lower than 10^−5^) and unspecific ones (archaeal sequences in bacterial amplicons and vice versa). This operation allowed us to reduce ASVs to 1111 for *Bacteria* and 563 for *Archaea*. By using the filtered ASVs, rarefaction curves were obtained for *Bacteria* and *Archaea* (Figure S2) and, according to them, rarefaction thresholds were set to 125,000 and 70,000, respectively. By using these thresholds, we were able to properly sample the observed biodiversity and retain all the samples.

**Figure 2:**
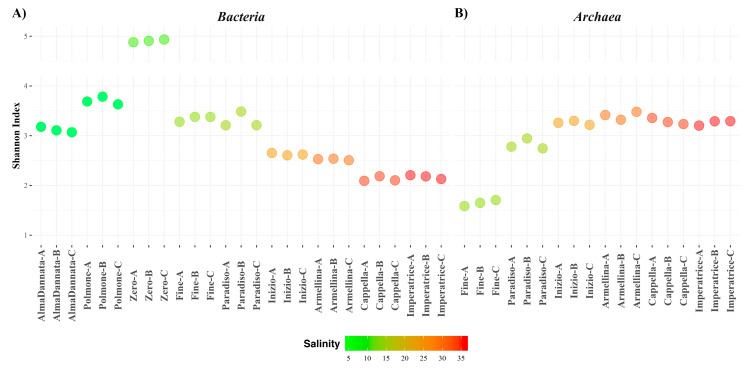
Shannon index inferred from 16S rRNA gene amplicon sequences for the assayed ponds of the MdS saltern. Ponds are plotted according to increasing salinity: from Alma Dannata to Imperatrice for *Bacteria* (**A**) and from Fine to Imperatrice for *Archaea* (**B**). The letters A, B and C accompanying the names of the ponds refer to the values of the experimental replicas. Salinity is expressed as salt percentage (w/v).

The alpha diversity was measured by using the Shannon Index and plotted as a dot-plot (Figure 2). The distribution of *Bacteria* showed an increase in Shannon values in the first three ponds (salinity ranging between 4.9 to 8.4%) and a continuous decrease from intermediate to high salinity values, indicating a negative influence of salinity on bacteria species evenness and richness. The high value observed for the pond with 8.4% salinity was possibly due to specific and currently unknown environmental and chemical-physical conditions, allowing a greater bacterial diversity (see also the taxonomic profiles). *Archaea* showed an opposite behavior, with an increase in the Shannon values from low to high salinity ponds. Statistically significant differences were evaluated by the Kruskal–Wallis test (*p* = 0 and 0.01 for *Bacteria* and *Archaea*, respectively) followed by the Dunn Test for pairwise comparisons (Table S3). In particular, according to the Shannon index distribution in *Bacteria*, the alpha diversity in Zero pond (the highest observed value) was statistically different compared to Alma Dannata and high salinity ponds (namely Inizio, Armellina, Cappella and Imperatrice). In *Archaea*, we observed statistically significant differences among the Fine pond (the lowest observed) and high salinity ponds.

**Figure 3:**
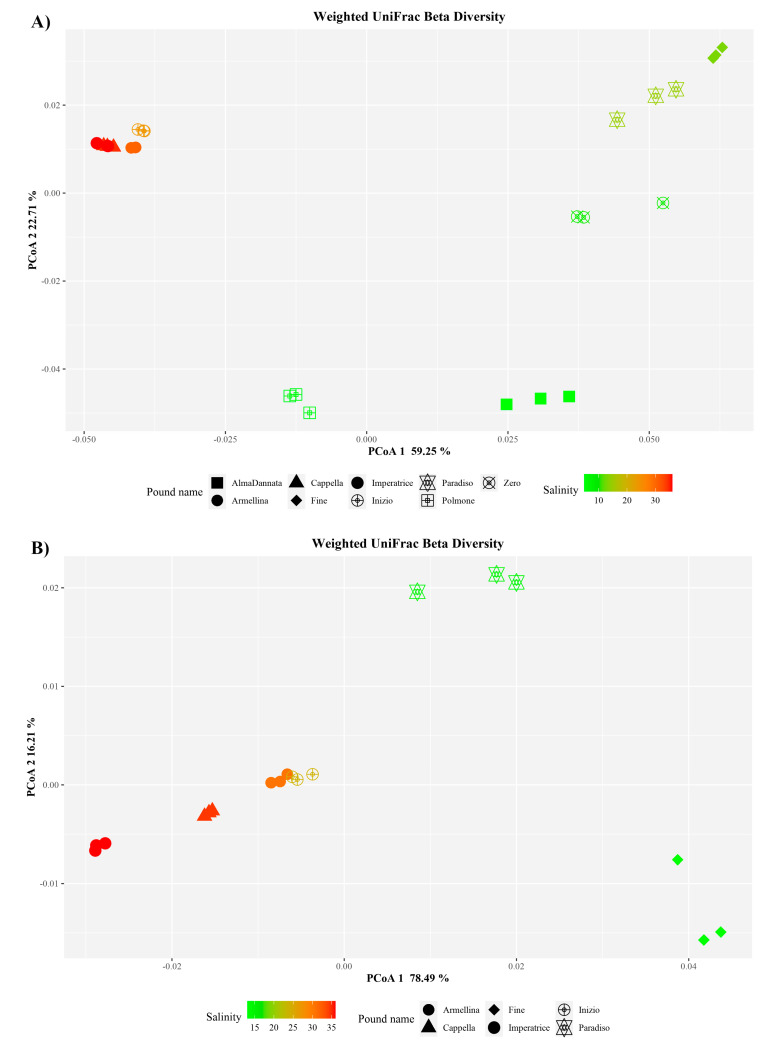
PCoA (Principal Coordinates Analysis) representations of weighted UniFrac analysis. (**A**) *Bacteria*; (**B**) *Archaea*. Salinity is reported as salt percentage (w/v).

Beta diversity was measured as weighted UniFrac and plotted as PCoA (Principal Coordinates analysis) (Figure 3). For both *Bacteria* and *Archaea*, it was possible to observe a clustering of samples on the first component (59.25 and 78.49%, respectively) that may be explained by salinity concentrations. PERMANOVA analysis confirmed these observations for both the measures. In particular, salinity explained about 42% (*p*-value = 0.001) and 69% (*p*-value = 0.001) of data variability in weighted UniFrac data for *Bacteria* and *Archaea*, respectively.

Pearson’s product moment correlation coefficient (PC) was used to measure the correlation between ASVs and salinity (Table S4). For *Bacteria*, 45 ASVs resulted correlated with salinity and, in particular, 33 positively (PC from 0.67 to 0.97) and 12 negatively (PC from −0.67 to −0.80). In total, 20 out of the 33 positively correlated ASVs were taxonomically classified as *Salinibacter*. For *Archaea*, 32 ASVs resulted positively correlated with salinity (PC from 0.81 to 0.97) and 36 ASVs were negatively correlated (PC from −0.82 to −0.93). A total of 31 out of the 32 positively correlated ASVs belonged to the order *Halobacteriales*. The class *Woesearchaeia* was the most represented in the negatively correlated ASVs.

### 3.3. Taxonomic Profiles

**Figure 4:**
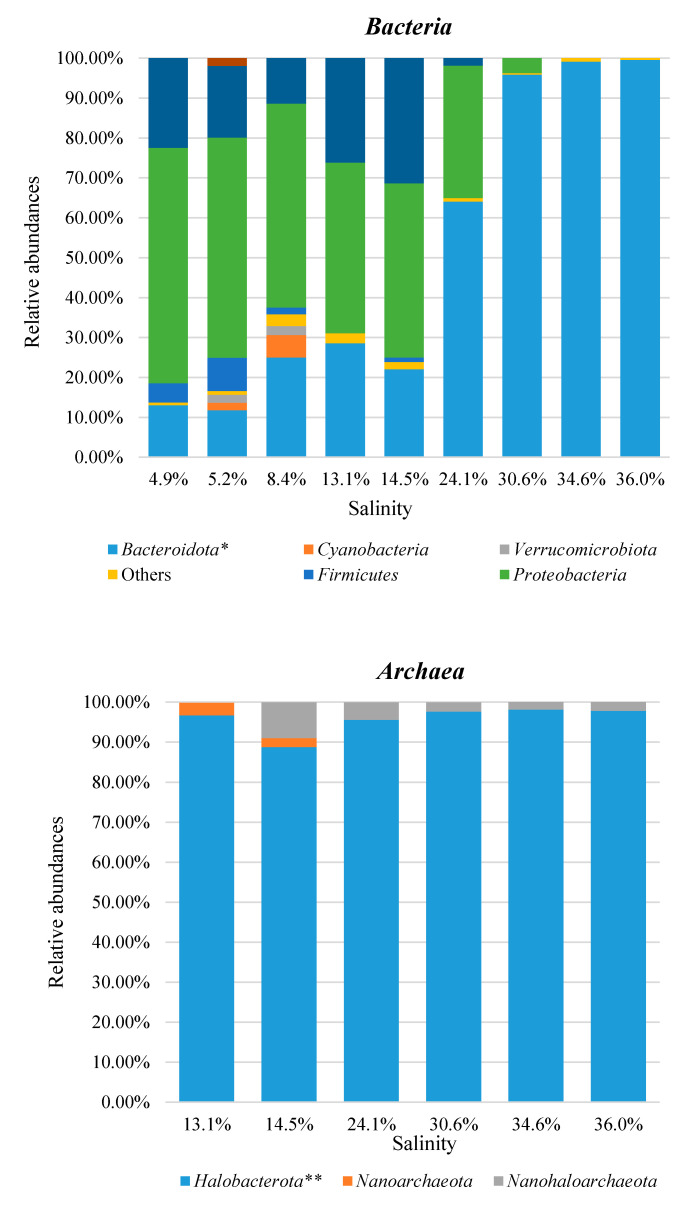
Bar charts of *Bacteria* and *Archaea* phyla identified at different salinities in MdS (Margherita di Savoia) saltern. Groups with relative abundances < 1.0% were joined as ‘Others’. Salinity is reported as salt percentage (w/v). * also known as *Bacteroidetes*; ** also known as *Euryarchaeota*.

**Figure 5:**
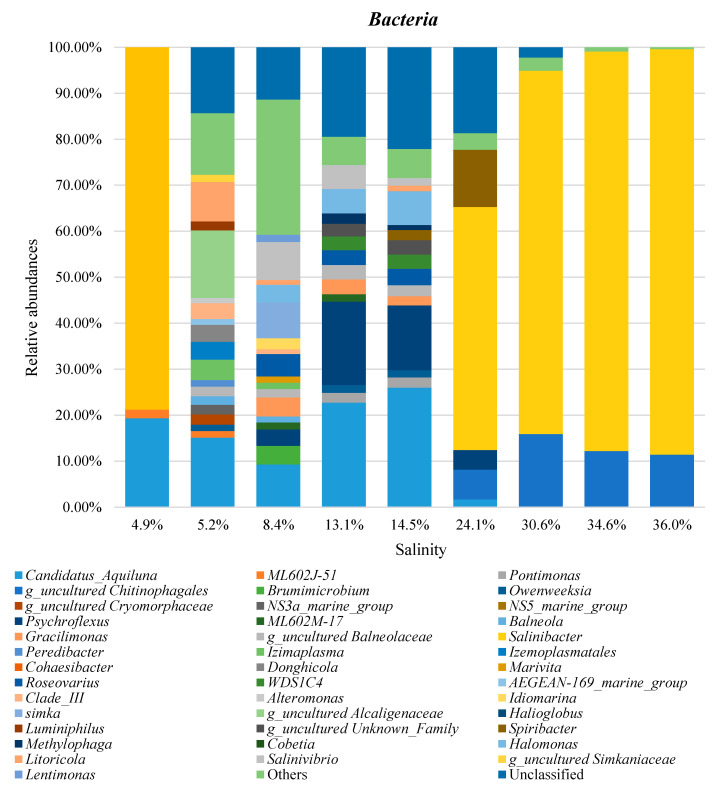

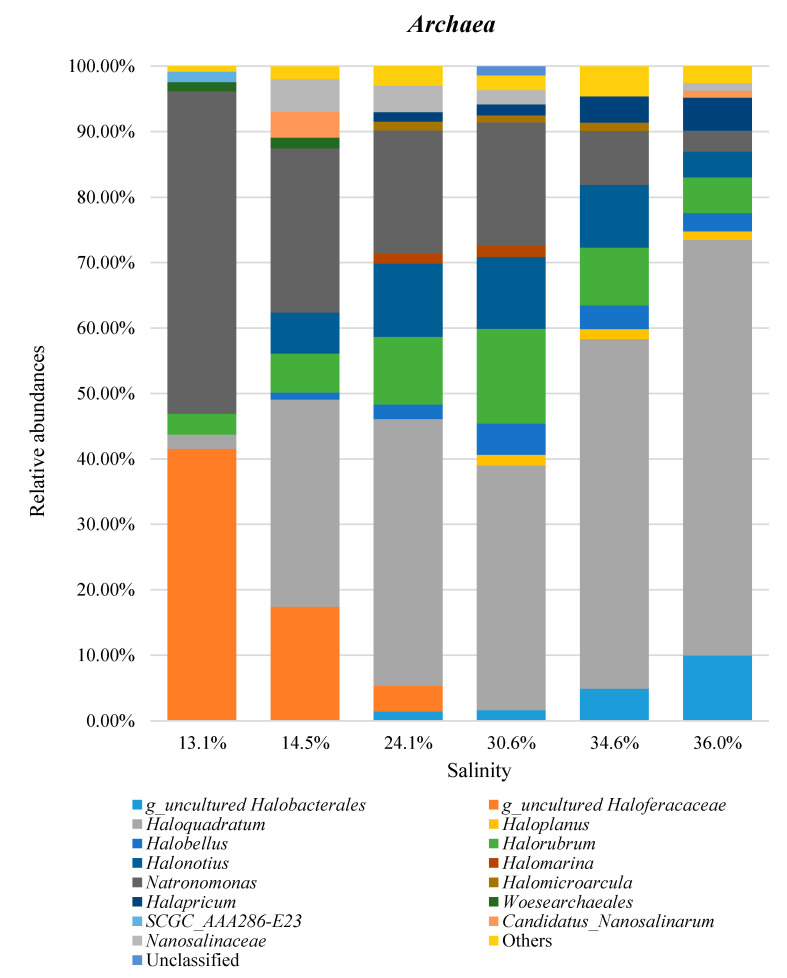
Bar charts of *Bacteria* and *Archaea* genera identified at different salinities in MdS saltern. Groups with relative abundances <1.0% were joined as ‘Others’. Salinity is reported as salt percentage (w/v). ASVs which could not be resolved at the genus level were reported with the notation g_uncultured followed by the name of the closest known parental rank. A list of the identified genera and their relative abundances is reported in Table S5.

The taxonomic distributions of *Bacteria* and *Archaea* are shown in Figure 4 for the phylum rank and in Figure 5 for the genus rank. All values were the average of the data from the three replicas.

Among *Bacteria*, *Proteobacteria* was the most abundant phylum in the evaporation ponds (up to 14.5% salinity). It reached percentages above 50% in waters at low salinity (4.9–8.4%), and then, it showed a progressive lowering, until disappearing at the highest salt concentrations (34.6–36.0%). The phylum *Actinobacteria* reached its maximum of relative abundance (31.35%) at the intermediate salinity of 14.5%. It dropped to 1.85% in the next pond (24.1% salinity) and it was negligible at higher salinities. Differently from these phyla, *Bacteroidota* abundance showed a steady increase with salinity, being the most represented phylum starting from 24.1% of salt concentration and reaching percentages above 95% in the three final ponds (30.6–36.0% salinity).

As for *Archaea,* our analysis identified a single predominant phylum (*Halobacterota*) throughout the tested waters (13.1–36.0% salinity). It is interesting to note the presence in these waters of the *Nanoarchaeota* and *Nanohaloarchaeota* phyla, known as components of the DPANN (*Diapherotrites*, *Parvarchaeota*, *Aenigmarchaeota*, *Nanoarchaeota*, *Nanohaloarchaeota*) superphylum [46]. Taken together, they reach a maximum of about 11% in the pond, with a salinity of 14.5%. DPANN lineages have generally been described in a variety of habitats worldwide including hypersaline environments and are characterized by smaller sizes (~ 0.6 vs. 2–4 10^−6^ m) and smaller genomes (< 1.0 vs. 3–4 Mb) than other archaeal cells [46,47].

At the genus level for *Bacteria*, the ponds at low and intermediate salinity of the evaporation zone (4.9–14.5%) showed major richness, which decreased with the increase in salinity. Additionally, in this case, the large salinity change from Paradiso (14.5% salinity) to Inizio (24.1% salinity) ponds had a remarkable influence on bacterial composition, reducing the number of genera from 16 to 7 (Figure 5).

Concerning the abundance of specific genera, *Salinibacter* was the genus showing the highest prevalence: 88% in the pond with 36.0% salinity. Other highly prevailing genera were “*Candidatus* Aquiluna” (26% of abundance at 14.5% salinity), *Psychroflexus* (18% of abundance at 13.1% salinity) and an unclassified genus of the *Chitinophagales* order (16% of abundance at 30.6% salinity). There were no other genera above 15% of abundance in the examined waters. *Salinibacter* (phylum *Bacteroidota*) is a known genus of solar salterns, diffused worldwide. Members of this genus are characterized by sharing many features with archaeal cells of the family *Halobacteriaceae* that live in the same habitat [48]. The *Psychroflexus* (phylum *Bacteroidota*) genus has also been described in saline environments, including marine solar salterns and graduation towers [49,50]. Little is known about the “*Candidatus* Aquiluna” genus (phylum *Actinobacteria*). The first species of this genus were isolated from four different freshwater habitats located in China, France, United Kingdom and Tanzania (the last described as a tropical site) [51]. It is a selenoid-shaped photoheterotroph organism, consisting of actinorhodopsin molecules for light energy transduction. After the first isolation, “*Candidatus* Aquiluna” members were identified in Arctic sea waters, and brackish ice brine [52,53,54,55] and Indian brackish coastal waters [56]. Notably, “*Candidatus* Aquiluna” was identified as the dominant phylotype in brackish ice brine, which has a lower salinity than immediate sub-ice seawater [55]. The presence of this genus in saltern waters has not been reported so far. “*Candidatus* Aquiluna”, *Psychroflexus* and *Salinibacter* were also the genera with the greatest ability to live at different concentrations of salts, being present in the salinity ranges of 4.9–24.1% (“*Candidatus* Aquiluna”), 8.4–24.1% (*Psychroflexus*), and 24.1–36.0% (*Salinibacter*). On the contrary, several genera (fourteen out of the forty reported in Figure 5) showed a limited capacity to adapt to different salt concentrations, being present only in single ponds of low salinity (4.9–8.4%) and with generally low abundances (1–4%).

Differently from *Bacteria*, the number of archaeal genera increased with salinity, reaching a maximum of eleven genera in the Inizio and Armellina ponds (24.1 and 30.6% salinity, respectively). Three genera (*Haloquadratum, Natronomonas* and *Halorubrum*) were present throughout the investigated ponds, with the first two reaching the highest relative abundances (*Haloquadratum* about 63% in the pond with 36.0% salinity; *Natronomonas* about 49% in the 13.1% salinity pond). The high presence of the *Haloquadratum* genus in crystallizer waters makes the archaeal profile of MdS saltern different from those described for the other two marine salterns located in the Adriatic sea (Secovlje in Slovenia and Ston in Croatia), in which the genus was not detected [12]. The square-shaped *Haloquadratum* cells had already been identified in the MdS saltern by phase-contrast microscopy [57].

The genus *Halorubrum* was first described about 25 years ago. Species of this genus are quite common in hypersaline habitats, including salterns, saline lakes and soils and salted foods [58]. It includes rods or pleomorphic cells forming red-orange pigmented colonies. It is the genus with the largest number of species among all genera of *Halobacteria*, although recent studies based on genome analysis highlighted the possibility that different species should actually be considered as single ones [59].

*Natronomonas* is a halophilic genus*,* deeply studied for possible biotechnological applications, for which only four species have been described so far [60]. The high abundance of this genus in waters of the saltern of MdS with low salinity (13.1%) differs from the optimal growth conditions currently reported (including salt concentrations around 25%) and leads to the hypothesis of the presence of new species.

The other identified genera showed a lower diffusion throughout the saltern and, with the exception of the unresolved genus ‘g_uncultured *Haloferacaceae*’ (with a relative abundance of about 42% in the 13.1% salinity pond), were generally in the 1–10% range.

The influence of salinity on microbiota composition was also clearly shown by heat maps (Figure S3). For both *Bacteria* and *Archaea*, microbial diversities seemed to be grouped according to ranges of salinities: low salinity (4.9–8.4% salinity), intermediate salinity (13.1–14.5% salinity), and high salinity (24.1–36.0% salinity).

### 3.4. Combined Prokaryotic Populations

**Figure 6:**
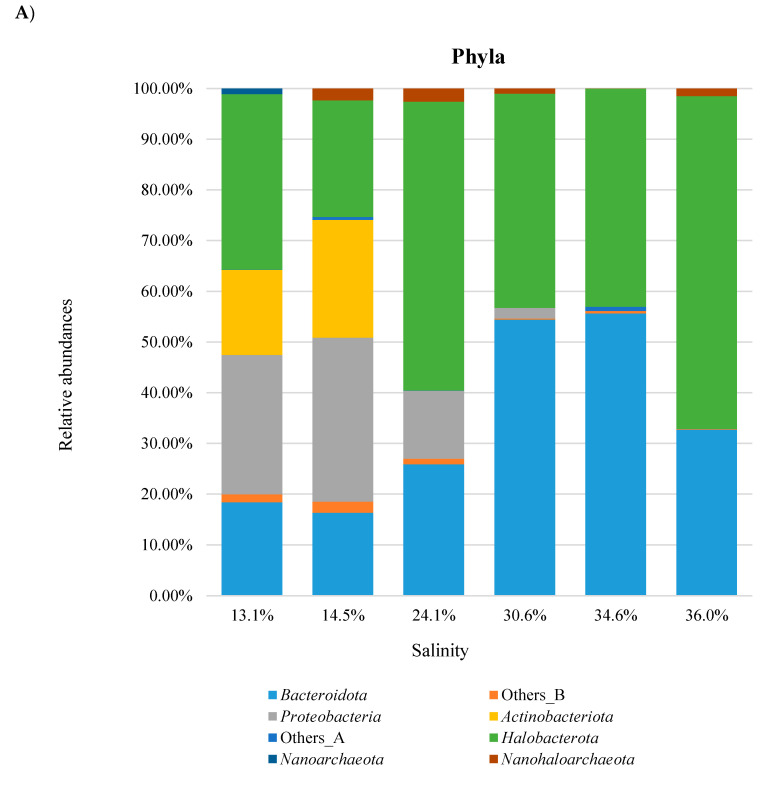

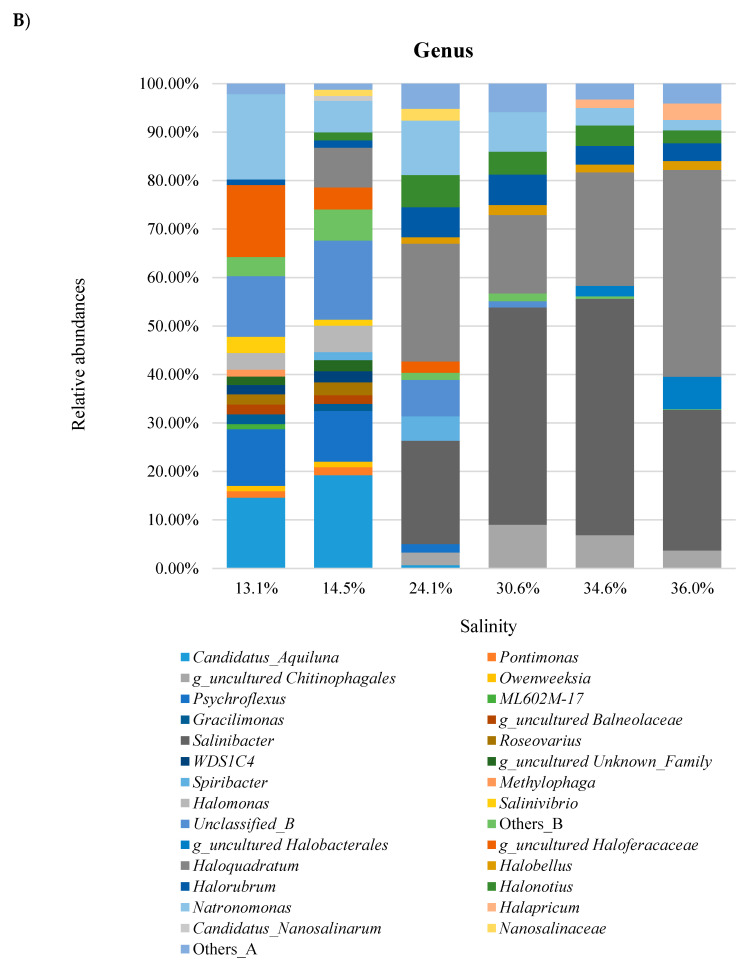
Bar charts of taxonomic classification of *Bacteria* and *Archaea* identified in MdS saltern. (**A**) phyla; (**B**) genera. Relative abundances (RA_i_) were obtained applying the formula RA_i_ = RA_i,m_*RA_D,cf_/100, where RA_i,m_ is the relative value obtained by metabarcoding analysis and RA_D,cf_ is the relative abundance of the parental domain obtained by CARD-FISH analysis. Groups with relative abundances < 1.0% were joined as ‘Others_B’ and ‘Others_A for *Bacteria* and *Archaea*, respectively. A list of all the identified genera and their relative abundances is reported in Table S6.

The availability of distinct metabarcoding data for *Bacteria* and *Archaea* and the description of microbial populations as obtained by DAPI and CARD-FISH analyses also allowed us to establish a *bona fide* profile of prokaryotic taxonomies for the waters at different salinity of the saltern of MdS (Figure 6). The evaluation was, however, only possible for the last six ponds (13.1–36.0% salinity), for which metabarcoding data were available for both *Archaea* and *Bacteria*. In practice, the relative abundance data for each of the two domains (provided by the metabarcoding analysis) were combined with each other, taking into account their relative abundances as obtained by the CARD-FISH analysis.

These data show that although the bacterial and archaeal diversities have an opposite correlation with salinity (the number of phyla and genera decreases for *Bacteria*, and increases for *Archaea*), the relative abundances of the two domains in each pond do not follow the same trend. Indeed, while *Archaea* was the prevailing domain in the ponds with salinities of 24.1 and 36.0%, *Bacteria* prevailed at salinities of 13.1, 14.5, 30.6 and 34.6%. The alternating prevalence between the two domains was mostly the effect of the high abundance in highly salted ponds of the *Salinibacter* genus. It was in absolute, the most abundant prokaryotic genus in the analyzed waters of all the saltern, reaching the highest count of 1.43·10^5^ cells/mL in the Armellina pond (30.6% salinity). Even in the most salted pond (Imperatrice, 36.0% salinity), the number of *Salinibacter* cells is comparable with that of the most represented *Archaea* genus (*Haloquadratum*) (4.14·10^4^ vs. 6.10·10^4^ cells/mL). These data suggest that the assumption that extreme hypersaline waters are dominated by *Archaea* [33] cannot be considered of general validity. These results also indicate that at the genus level, although salinity has some influence on microbial diversity, it does not exert a negative effect on cell viability.

**Figure 7:**
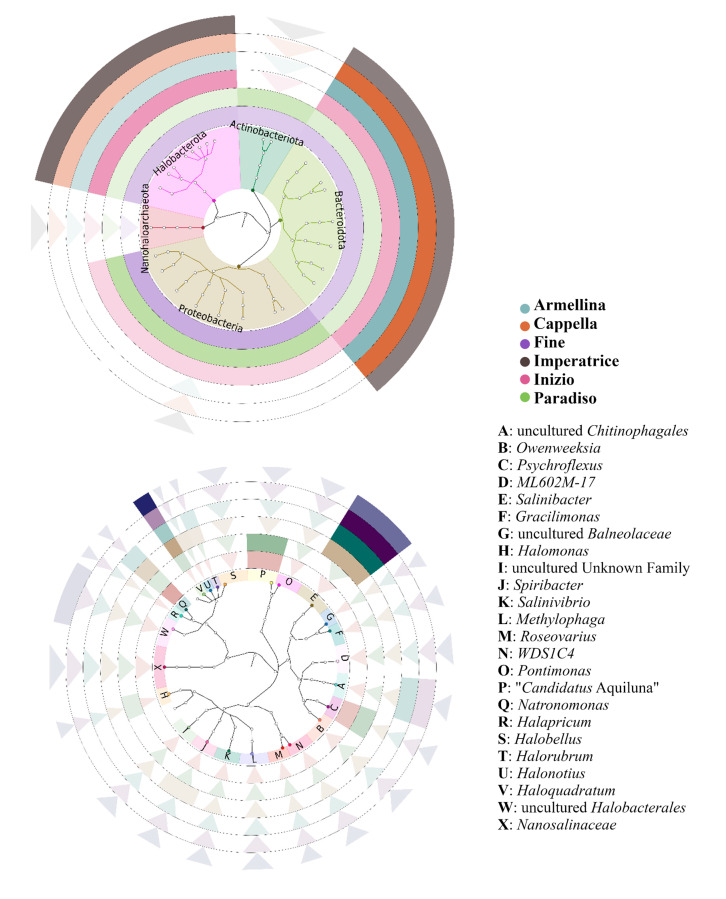
Phylogenetic graphs correlating identified phyla and genera and their abundances. Inside each graph, taxonomic trees are reported with full circles to indicate identified phyla (above) and genera (below). Names of phyla are reported inside the graph; names of genera are indicated in the side legend. Ponds are represented by external rings corresponding (from inside to outside) to increasing salinity. Their names are reported in the side legend.

A graphical representation of the taxonomic tree of the most abundant lineages (i.e., ≥1%) in the ponds of the MdS saltern and their relative abundances was obtained by the GraPhlAn tool [45] and is reported in Figure 7. Abundances of single lineages in specific ponds were shown according to a 0 to 1 scale of transparency and were reduced to inward pointing arrows when the transparency value was below 0.1. These graphs take into account both the CARD-FISH and the metabarcode results and summarize the prokaryotic profiles of the six ponds with salinity from 13.1 to 36.0%.

At the phylum level, only the *Bacteroidota* and *Halobacterota* phyla are present with relevant abundances (full colored sections) in all the ponds. Interestingly, the two phyla show alternating prevalence in the different ponds (see color intensities), which can be explained on the basis of the presence of specific genera (see below). Other phyla with relevant abundances are *Actinobacteriota*, in the two evaporation ponds Fine (salinity 13.1%) and Paradiso (salinity 14.5%), and *Proteobacteria* in the two evaporation ponds and in the first crystallizer pond Inizio (salinity 24.1%). The *Nanohaloarchaeota* phylum is present in the six ponds, but with relatively low abundances.

The genera graph shows that *Salinibacter* (*Bacteroidota*) and *Haloquadratum* (*Halobacterota*) are the two prevailing genera in the four crystallizer ponds (salinity 24.1–36.0%) and explains how their relative abundances influence the abundance of the parental phyla. Indeed, in the Imperatrice pond (salinity 36.0%, outermost ring), it is possible to observe a higher abundance of *Haloquadratum* than *Salinibacter*. Conversely, in the Cappella and Armellina ponds (salinity 34.6 and 30.6%, respectively), *Salinibacter* prevails over *Haloquadratum*. In the Inizio pond (salinity 24.1%), *Haloquadratum* is again more abundant than *Salinibacter*. A minor contribution to the phyla abundances in these ponds is also given by an unclassified genus of the *Chitinophagales* order (*Bacteroidota*). In the two evaporation ponds, other genera become relevant, such as “*Candidatus* Aquiluna”, *Psychroflexus*, *Spiribacter* and *Halomonas* for *Bacteria* and *Natronomonas* for *Archaea*.

Although the dynamic of microbiota composition in the MdS saltern showed common features with other salterns, it is difficult to make quantitative comparisons. This is mainly due to the different investigative approaches. For example, in the case of other salterns located on the Adriatic Sea (Secovlje in Slovenia and Ston in Croatia), the identification of prokaryote profiles was carried out on the basis of the analysis of 16S rRNA gene amplicon libraries developed in *Escherichia coli* [12]. This different approach may explain the great difference observed for the *Halorubrum* genus, found with a maximum abundance of 14.47% in the MdS saltern, and with abundances of 66 and 87% in Secovlje and Ston salterns, respectively.

The microbiota compositions of the MdS saltern also show quantitative differences compared to those of the saltern of Santa Pola (Spain), the only saltern in the Mediterranean area for which an NGS-based analysis of prokaryotes was performed [61,62]. The Santa Pola multipond solar saltern is one of the most studied hypersaline environments, located about 20 km south from Alicante on the Spanish Mediterranean Sea coast. Ponds are at increasing saline concentrations from 13% up to 37%. Deep shotgun sequencing of DNA extracted from the different ponds was carried out by the pyrosequencing approach. A taxonomic profile of prokaryotes was then established by analyzing the sequencing data for the content of the 16S rRNA genes. The microbiota composition of these salterns is quantitatively different from that of MdS saltern, even at the high rank levels of domain and phylum. Indeed, while in Santa Pola saltern, *Archaea* relative abundance is around 27% in the intermediate 13% salinity pond, and reaches values around 90% in the crystallizer pond at 33.37% of salinity, in MdS saltern, it is 35.77% in the 13.1% pond and reaches 43.87% in the crystallizer pond at 34.6% salinity (it must be underlined that at the domain level, the percentages for the MdS saltern were derived solely from the CARD-FISH data). At the phylum level, the most striking difference is that concerning *Bacteroidota* which, even if always present, show different values and trends in the two salterns. In Santa Pola saltern, the relative abundance of this phylum reaches a maximum of 15% in ponds with intermediate salinity (19%) and has a moderate decrease to 9–10% in crystallizer ponds. In MdS ponds, *Bacteroidota* is always more abundant than in Santa Pola counterparts. Its relative abundance goes from 15–18% in evaporation ponds (13.1 and 14.5% salinity) to more than 50% in crystallizer ponds of 30.6 and 43.6% salinity. In both the salterns, even if at different relative abundances, the other most represented phyla of *Bacteria*, *Proteobacteria* and *Actinobacteria*, show a similar decreasing trend as salinity increases. *Archaea* are almost exclusively represented by the *Halobacterota* phylum in both the salterns.

The genera with the highest abundance in the high-salinity ponds of MdS are *Salinibacter* (48.76% at 34.6% salinity) and *Haloquadratum* (42.64% at 36.0% salinity), differing from what observed for Santa Pola, where the most abundant genus in crystallizer ponds is *Haloquadratum* (with abundances between 29.5 and 58.0%), and *Salinibacter* is only in the 4.7–9.1% range. *Halorubrum* in Santa Pola reaches an abundance of 23.1% in the crystallizer pond at 33% salinity. In MdS saltern it reaches a maximum of 6.26% of abundance at 30.5% salinity.

Whether these differences are due to the different geographical and environmental conditions of the two salterns and/or to the different analytical approach (shotgun sequencing vs. amplicon sequencing) cannot be established with available data. More uniform analysis will be necessary to clarify this point.

## 4. Conclusions

A DNA metabarcoding analysis of prokaryotic populations in the waters of the saltern of MdS was carried out by using distinct pairs of primers for the amplification of bacterial and archaeal 16S rRNA gene regions. CARD-FISH cell count corroborated metabarcoding analysis and allowed us to obtain a combined profile of prokaryotic taxonomies at different salinities. The analysis showed some expected changes in the bacterial and archaeal composition along the investigated range of salinity (4.9–36.0%), but also revealed some specific features which add new details on not yet exhaustively studied microbes. This was the case of the bacterial genus “*Candidatus* Aquiluna”, known as typical of fresh and sea waters, which was detected with a relative abundance of about 21% at 14.5% salinity. Of interest also was the finding of the archaeal genus *Natronomonas* with an abundance of about 18% at relatively low salinity value (13.1%), since the few cultivated species are grown at a salinity of 25%. Another interesting feature of the MdS saltern was related to the distribution of *Archaea* in a wide range of salinities, as demonstrated by CARD-FISH analysis. Archaeal cells were detected starting from waters at low salinity (22.5% of relative abundance within prokaryotes in the Alma Dannata pond with salinity of 4.9%) up to the crystallizer waters, where they reached abundances ranging between 43–67%. It should be noted, however, that in crystallizer waters, bacterial and archaeal cells showed abundances with alternating prevalence. This largely depends on the high number of cells of the bacterial *Salinibacter* genus detected at high salinities.

A comparison with other solar salterns of the same area (the Adriatic and the Mediterranean Seas) showed distributions of prokaryotes only partially similar to those of the MdS saltern. Nevertheless, establishing common traits and differences of bacterial composition in the marine solar salterns spread around the world, or located in a specific area as the Mediterranean Sea, can be of great biological and ecological interest. NGS techniques are undoubtedly well suited to address this objective, once common investigative approaches will be adopted.

## Figures and Tables

**Table 1:** Names and properties of collected water samples.

Pond Name	Salinity (% w/v)	T (°C)	pH
**Alma Dannata**	4.9	24	8.55
**Polmone**	5.2	26	8.62
**Zero**	8.4	27	8.07
**Fine**	13.1	25	7.77
**Paradiso**	14.5	27	7.73
**Inizio**	24.1	29	7.59
**Armellina**	30.6	30	7.46
**Cappella**	34.6	30	7.27
**Imperatrice**	36.0	30	7.20

## Supplementary Materials

The following are available online at https://www.mdpi.com/2076-2607/8/6/936/s1, Table S1, List of primers. Table S2, Cell counts (cells/mL) obtained from the different waters of the saltern of MdS. Table S3, Summary of pairwise Dunn tests. Table S4, Pearson’s product moment correlation coefficient (PC) between ASVs and salinity. Table S5, Relative abundances of bacterial and archaeal genera obtained by metabarcoding analysis. Table S6, Relative abundances of bacterial and archaeal genera obtained by combination of metabarcoding and CARD-FISH data. Figure S1, Detection of bacteria and archaea cells by CARD-FISH analysis. Figure S2, Rarefaction curves of 16S rRNA ASVs for the assayed ponds of the MdS saltern. Figure S3, Heat maps of microbial amplicons similarity matrix of MdS ponds.
