# Supplementary material for: A Differential Metabarcoding Approach to Describe Taxonomy Profiles of Bacteria and Archaea in the Saltern of Margherita di Savoia (Italy)
Source: Microorganisms. 2020 Jun 22;8(6):936. doi: 10.3390/microorganisms8060936 (PMC7356649; doi:10.3390/microorganisms8060936)
Supplement: Supplementary file 1 [file microorganisms-08-00936-s001.pdf]

SUPPLEMENTARY MATERIAL

A differential metabarcoding approach to describe taxonomy profiles of Bacteria and Archaea in the Saltern of Margherita di Savoia (Italy)

Claudia Leoni[1,a], Mariateresa Volpicella[2,a,*], Bruno Fosso[1,a], Caterina Manzari[1], Elisabetta Piancone[2], Maria C.G. Dileo[2], Erika Arcadi[3], Michail Yakimov[4], Graziano Pesole[1,2] and Luigi R. Ceci[1,*]

[1]Institute of Biomembranes, Bioenergetics and Molecular Biotechnologies, CNR, Via Amendola 122/O, 70126 Bari, Italy; [2]Department of Biosciences, Biotechnologies and Biopharmaceutics, University of Bari "Aldo Moro", Via Orabona 4, 70126 Bari, Italy; [3]Stazione Zoologica Anton Dohrn, Via dei Mille 46, 98057 Milazzo (Messina), Italy; [4]Institute for Biological Resources and Marine Biotechnology, CNR, Spianata San Raineri, 86 - 98122 Messina, Italy.

[a] These Authors have contributed equally to this work.

*Correspondence:
Mariateresa Volpicella: mariateresa.volpicella@uniba.it
Luigi R. Ceci: l.ceci@ibiom.cnr.it.

List of material:

Table S1. **List of primers.**
Table S2. **Cell counts (cells/ml) obtained from the different waters of the saltern of MdS.**
Table S3. **Summary of pairwise Dunn tests.**
Table S4. **Pearson's product moment correlation coefficient (PC) between ASVs and salinity.**
Table S5. **Relative abundances of bacterial and archaeal genera obtained by metabarcoding analysis.**
Table S6. **Relative abundances of bacterial and archaeal genera obtained by combination of metabarcoding and CARD-FISH data.**

Figure S1. **Detection of bacteria and archaea cells by CARD-FISH analysis.**
Figure S2. **Rarefaction curves of 16S rRNA ASVs for the assayed ponds of the MdS saltern.**
Figure S3. **Heat maps of microbial amplicons similarity matrix of MdS ponds.**

Table S1. List of primers for PCR and probes for CARD-FISH.

Name	Sequence (5'-3')	Ref.
Eub338 I	GCTGCCTCCCGTAGGAGT	23,24
Eub338 II	GCAGCCACCCGTAGGTGT	23,24
Eub338 III	GCTGCCACCCGTAGGTGT	23,24
Arch915	GTGCTCCCCCGCCAATTCCT	25
B-V5*	TCGTCGGCAGCGTCAGATGTGTATAAGAGACAG/ATTAGATACCCYGGTAGTCC	28,29
A-V6*	GTCTCGTGGGCTCGGAGATGTGTATAAGAGACAG/ACGAGCTGACGACARCCATG	28,29
Arch_349F*	TCGTCGGCAGCGTCAGATGTGTATAAGAGACAG/GYGCASCAGKCGMGAAW	30
Arch_806R*	GTCTCGTGGGCTCGGAGATGTGTATAAGAGACAG/GGACTACVSGGGTATCTAAT	30

* The first part of the primer, before the slash, corresponds to the Nextera transposase sequence, required by the Illumina protocol.

Symbols for degenerate nucleotides are according to the IUPAC nomenclature: Y:—C/T; R:—A/G; S:—G/C; K:—G/T; M:—A/C; W—:A/T; V—:A/C/G.

Table S2. Cell counts (cells/ml) obtained from the different waters of the saltern of MdS. Ponds are indicated by conventional names, with salinity reported as salt percentage (w/v). Three different counts were carried out for each pond. B/T, A/T and (B+A)/T are the relative abundances of bacterial (B), archaeal (A) and living prokaryotic cells (B + A), respectively, compared to total cells (T) as counted by DAPI staining.

	**DAPI**	***Bacteria***	***Archaea***	**DAPI dev**	***Bacteria* dev**	***Archaea* dev**	**B/T (%)**	**A/T (%)**	**B/ (B+A) (%)**	**A/ (B+A) (%)**	**(B+A)/T (%)**
**Alma Dannata (4.9%)**	**2.70E+05**	**5.04E+04**	**1.46E+04**	**3.74E+04**	**5.80E+03**	**3.43E+03**	**18.7**	**5.4**	**77.5**	**22.5**	**24.1**
**Polmone (5.2%)**	**3.11E+05**	**2.70E+05**	**3.51E+04**	**1.12E+04**	**1.48E+04**	**3.85E+03**	**86.8**	**11.3**	**88.5**	**11.5**	**98.1**
**Zero (8.4%)**	**2.09E+05**	**1.93E+05**	**1.31E+04**	**1.04E+04**	**7.39E+03**	**1.77E+03**	**92.3**	**6.3**	**93.6**	**6.4**	**98.6**
**Fine (13.1%)**	**3.10E+05**	**1.94E+05**	**1.08E+05**	**1.76E+04**	**2.05E+04**	**2.92E+03**	**62.6**	**34.8**	**64.2**	**35.8**	**97.4**
**Paradiso (14.5%)**	**5.06E+05**	**3.60E+05**	**1.26E+05**	**3.10E+04**	**7.93E+03**	**1.24E+04**	**71.1**	**24.9**	**74.1**	**25.9**	**96.0**
**Inizio (24.1%)**	**2.80E+05**	**1.07E+05**	**1.58E+05**	**1.04E+04**	**1.02E+04**	**1.36E+04**	**38.2**	**56.4**	**40.4**	**59.6**	**94.6**
**Armellina (30.6%)**	**3.42E+05**	**1.81E+05**	**1.38E+05**	**2.00E+04**	**2.09E+04**	**9.73E+03**	**52.9**	**40.4**	**56.7**	**43.3**	**93.3**
**Cappella (34.6%)**	**2.47E+05**	**1.24E+05**	**9.69E+04**	**1.20E+04**	**1.06E+04**	**1.04E+04**	**50.2**	**39.2**	**56.1**	**43.9**	**89.4**
**Imperatrice (36.0%)**	**1.84E+05**	**4.70E+04**	**9.60E+04**	**1.16E+04**	**5.32E+03**	**1.04E+04**	**25.5**	**52.2**	**32.9**	**67.1**	**77.7**

Table S3. Summary of pairwise Dunn tests. Pond names and salinities are listed in columns and rows. The inferred adjusted p-values are shown. Significant p-values are in bold and starred (*≤ 0.05, **≤ 0.01, ***≤0.001).

***Bacteria*:**

**Pond (Salinity %)**	**Fine (13.1%)**	**Paradiso (14.5%)**	**Inizio (24.1%)**	**Armellina (30.6%)**	**Cappella (34.6%)**	**Imperatrice (36.0%)**	**Alma Dannata (4.9%)**	**Polmone (5.2%)**
**Paradiso (14.5%)**	0.4387							
**Inizio (24.1%)**	0.1085	0.1400						
**Armellina (30.6%)**	**0.0448***	0.0614	0.3217					
**Cappella (34.6%)**	**0.0059****	**0.0090****	0.0992	0.2053				
**Imperatrice (36.0%)**	**0.0118***	**0.0175***	0.1518	0.2858	0.2858			
**Alma Dannata (4.9%)**	0.2202	0.2685	0.3217	0.1773	**0.0402***	0.0679		
**Polmone (5.2%)**	0.2685	0.2202	**0.0320***	**0.0103***	**0.0009*****	**0.0020****	0.0825	
**Zero (8.4%)**	0.1400	0.1085	**0.0103***	**0.0027****	**0.0002*****	**0.0004*****	**0.0320***	0.3217

***Archaea*:**

**Pond (Salinity %)**	**Fine (13.1%)**	**Paradiso (14.5%)**	**Inizio (24.1%)**	**Armellina (30.6%)**	**Cappella (34.6%)**
**Paradiso (14.5%)**	0.2456				
**Inizio (24.1%)**	**0.0234***	0.0968			
**Armellina (30.6%)**	**0.0004*****	**0.0037****	0.0843		
**Cappella (34.6%)**	**0.0109***	0.0541	0.3798	0.1422	
**Imperatrice (36.0%)**	**0.0234***	0.0968	0.5000	0.0843	0.3798

Table S4. Pearson's product moment correlation coefficient (PC) between ASVs and salinity.

Bacteria:

ASV	Pearson Correlation	P-value	taxonomy
aa1f4fd53f406a3613fcf5661e0b8035	0.97	0.000	d__*Bacteria;*p__*Bacteroidota;*c__*Rhodothermia;*o__*Rhodothermales;*f__*Rhodothermaceae;*g__*Salinibacter*
99c793755e7c20d3416d3e113d63475e	0.96	0.000	d__*Bacteria;*p__*Bacteroidota;*c__*Rhodothermia;*o__*Rhodothermales;*f__*Rhodothermaceae;*g__*Salinibacter*
da3ae34876ceb295b8846f57e0bf98fc	0.96	0.000	d__*Bacteria;*p__*Bacteroidota;*c__*Rhodothermia;*o__*Rhodothermales;*f__*Rhodothermaceae;*g__*Salinibacter*
b7c04eaa669a35ed42a78381dfc78138	0.96	0.000	d__*Bacteria;*p__*Bacteroidota;*c__*Rhodothermia;*o__*Rhodothermales;*f__*Rhodothermaceae;*g__*Salinibacter*
2c815d6b32f92298b7578702b0dd1510	0.96	0.000	d__*Bacteria;*p__*Bacteroidota;*c__*Rhodothermia;*o__*Rhodothermales;*f__*Rhodothermaceae;*g__*Salinibacter*
be1f4e92c0ef762f0a1b05758e0a4bf9	0.95	0.000	d__*Bacteria;*p__*Bacteroidota;*c__*Rhodothermia;*o__*Rhodothermales;*f__*Rhodothermaceae;*g__*Salinibacter;*s__uncultured *Salinibacter*
3a3161d5e80646e5938102d9d23acf5f	0.95	0.000	d__*Bacteria;*p__*Bacteroidota;*c__*Bacteroidia;*o__*Chitinophagales;*f__uncultured *Chitinophagales;*g__uncultured *Chitinophagales;*s__uncultured *Chitinophagales*
34b2ada6eb11c95621549b76a674c1c1	0.94	0.000	d__*Bacteria;*p__*Bacteroidota;*c__*Bacteroidia;*o__*Chitinophagales;*f__uncultured *Chitinophagales;*g__uncultured *Chitinophagales;*s__uncultured *Chitinophagales*
2119951188cda0bdd84b64568e593a4a	0.94	0.000	d__*Bacteria;*p__*Bacteroidota;*c__*Rhodothermia;*o__*Rhodothermales;*f__*Rhodothermaceae;*g__*Salinibacter*
c47adbe8321dce0f73d55e94e3e72780	0.93	0.000	d__*Bacteria;*p__*Bacteroidota;*c__*Rhodothermia;*o__*Rhodothermales;*f__*Rhodothermaceae;*g__*Salinibacter;*s__*Salinibacter_ruber*
e5936cfb1715b6b9b7e14c4f953f94d3	0.92	0.000	d__*Bacteria;*p__*Bacteroidota;*c__*Bacteroidia;*o__*Chitinophagales;*f__uncultured *Chitinophagales;*g__uncultured *Chitinophagales;*s__uncultured *Chitinophagales*
b1189c366efdb76647929e59bc67f874	0.92	0.000	d__*Bacteria;*p__*Bacteroidota;*c__*Bacteroidia;*o__*Chitinophagales;*f__uncultured *Chitinophagales;*g__uncultured *Chitinophagales;*s__uncultured *Chitinophagales*
57f64b6d2bae0c9eeed6063e20153acd	0.91	0.001	d__*Bacteria;*p__*Bacteroidota;*c__*Bacteroidia;*o__*Chitinophagales;*f__uncultured *Chitinophagales;*g__uncultured *Chitinophagales;*s__uncultured *Chitinophagales*
ac3da6eb5e5938d0b631004834859280	0.88	0.002	d__*Bacteria;*p__*Bacteroidota;*c__*Rhodothermia;*o__*Rhodothermales;*f__*Rhodothermaceae;*g__*Salinibacter;*s__uncultured *Salinibacter*
23c128e062a85e3f9edad481d971abb5	0.86	0.003	d__*Bacteria;*p__*Bacteroidota;*c__*Rhodothermia;*o__*Rhodothermales;*f__*Rhodothermaceae;*g__*Salinibacter;*s__uncultured *Salinibacter*
c2962efec2da9314edd38d7167b6aaea	0.86	0.003	d__*Bacteria;*p__*Bacteroidota;*c__*Rhodothermia;*o__*Rhodothermales;*f__*Rhodothermaceae;*g__*Salinibacter*
0d88996a0249a67e3208cb890f339219	0.81	0.009	d__*Bacteria;*p__*Bacteroidota;*c__*Rhodothermia;*o__*Rhodothermales;*f__*Rhodothermaceae;*g__*Salinibacter;*s__uncultured *Salinibacter*
2cd0e6986ba98bb71bc7cc679fd6e2c6	0.80	0.010	d__*Bacteria;*p__*Bacteroidota;*c__*Rhodothermia;*o__*Rhodothermales;*f__*Rhodothermaceae;*g__*Salinibacter*
49ef03099317acbf97e22e34a253fcb2	0.79	0.011	d__*Bacteria;*p__*Bacteroidota;*c__*Rhodothermia;*o__*Rhodothermales;*f__*Rhodothermaceae;*g__*Salinibacter*
3092c2440ff9a90461054bf40642f489	0.79	0.011	d__*Bacteria;*p__*Bacteroidota;*c__*Rhodothermia;*o__*Rhodothermales;*f__*Rhodothermaceae;*g__*Salinibacter;*s__uncultured *Salinibacter*
00a224b16122e353591ac23cc72d6e7d	0.78	0.013	d__*Bacteria;*p__*Proteobacteria;*c__*Gammaproteobacteria;*o__*Nitrococcales;*f__*Nitrococcaceae;*g__uncultured *Nitrococcaceae;*s__uncultured *Nitrococcaceae*
eaed4b8971f40866bd1f31e062f579f9	0.77	0.016	d__*Bacteria;*p__*Bacteroidota;*c__*Rhodothermia;*o__*Rhodothermales;*f__*Rhodothermaceae;*g__*Salinibacter;*s__uncultured *Salinibacter*
038e1e6407a6fbf106d92d05eb71621d	0.74	0.022	d__*Bacteria;*p__*Proteobacteria;*c__*Alphaproteobacteria;*o__*Rhodobacterales;*f__*Rhodobacteraceae;*g__*Sediminimonas;*s__uncultured *Sediminimonas*

0869c07ab138130212f6a46f19029879	0.74	0.024	d__Bacteria;p__Desulfobacterota;c__Desulfuromonadia;o__Bradymonadales;f__Bradymonadaceae;g__Bradymonadaceae;s__uncultured Bradymonadaceae
d4d4a7b2415a45986d9a4418acbff493	0.73	0.025	d__Bacteria;p__Desulfobacterota;c__Desulfuromonadia;o__Bradymonadales;f__Bradymonadaceae;g__Bradymonadaceae;s__uncultured Bradymonadaceae
fc3010d03af677da6c45d7ca8c5f6d1e	0.71	0.032	d__Bacteria;p__Bacteroidota;c__Bacteroidia;o__Chitinophagales;f__uncultured Chitinophagales;g__uncultured Chitinophagales;s__uncultured Chitinophagales
0ec4c750eed1d8de856d341ea479b427	0.71	0.034	d__Bacteria;p__Bacteroidota;c__Rhodothermia;o__Rhodothermales;f__Rhodothermaceae;g__Salinibacter;s__uncultured Salinibacter
1c45f7a1f3d3061ed0f29a04908f348b	0.70	0.036	d__Bacteria;p__Desulfobacterota;c__Desulfuromonadia;o__Bradymonadales;f__Bradymonadaceae;g__Bradymonadaceae;s__uncultured Bradymonadaceae
a4436f7093a0e7cafbb276baddf40e3a	0.69	0.039	d__Bacteria;p__Bacteroidota;c__Rhodothermia;o__Rhodothermales;f__Rhodothermaceae;g__Salinibacter
7512cf96f90d72ea620a31db72947a13	0.69	0.040	d__Bacteria;p__Bacteroidota;c__Rhodothermia;o__Rhodothermales;f__Rhodothermaceae;g__Salinibacter
00cd9e7bb10f47a21b0e61c54fa18bc6	0.69	0.040	d__Bacteria;p__Bacteroidota;c__Bacteroidia;o__Chitinophagales;f__uncultured Chitinophagales;g__uncultured Chitinophagales;s__uncultured Chitinophagales
f36dec1f57e22cf290c271114e3fc2e2	0.68	0.045	d__Bacteria;p__Proteobacteria;c__Alphaproteobacteria;o__Kiloniellales;f__Fodinicurvataceae;g__Rhodovibrio
1a99e469d7cc0789a1247729c7bc2166	0.67	0.049	d__Bacteria;p__Bacteroidota;c__Rhodothermia;o__Rhodothermales;f__Rhodothermaceae;g__Salinibacter;s__uncultured Salinibacter
4748d319246942d0f227de0f249b89fb	-0.67	0.050	d__Bacteria;p__Actinobacteriota;c__Actinobacteria;o__Micrococcales;f__Microbacteriaceae;g__Candidatus_Aquiluna;s__uncultured Candidatus_Aquiluna
8abac3ecc5613a43e2db3016c8231957	-0.68	0.044	d__Bacteria;p__Proteobacteria;c__Gammaproteobacteria;o__Oceanospirillales;f__Litoricolaceae;g__Litoricola;s__uncultured Litoricola
5aecc6ad4f49b8ed8b0fe71d086294c2	-0.68	0.044	d__Bacteria;p__Actinobacteriota;c__Actinobacteria;o__Micrococcales;f__Microbacteriaceae
7988d516d5c461335aec989e6e9f6678	-0.69	0.041	d__Bacteria;p__Bacteroidota;c__Bacteroidia;o__Flavobacteriales;f__Flavobacteriaceae;g__Psychroflexus;s__uncultured Psychroflexus
487af4ed0c1c3666a40833f83e10ecae	-0.69	0.040	d__Bacteria;p__Proteobacteria;c__Alphaproteobacteria;o__Rhodobacterales;f__Rhodobacteraceae
459af99af321115ac4423547dde3f5f5	-0.71	0.034	d__Bacteria;p__Actinobacteriota;c__Actinobacteria;o__Micrococcales;f__Microbacteriaceae;g__ML602J-51;s__uncultured ML602J-51
4ba2d372a67c34ba6b9e53c2f8a0dd60	-0.73	0.027	d__Bacteria;p__Actinobacteriota;c__Actinobacteria;o__Micrococcales;f__Microbacteriaceae;g__Candidatus_Aquiluna
76357b935a80b43cb75d98be6bb3e020	-0.73	0.026	d__Bacteria;p__Proteobacteria;c__Alphaproteobacteria;o__Rhodobacterales;f__Rhodobacteraceae;g__Marivita
685246984508b28064b88baeac9f3864	-0.76	0.017	d__Bacteria;p__Proteobacteria;c__Gammaproteobacteria;o__Alteromonadales;f__Alteromonadaceae;g__Alteromonas;s__Alteromonas_mediterranea
d96b8ae4478e8036ffcac7d0ac078c3c	-0.77	0.015	d__Bacteria;p__Proteobacteria;c__Gammaproteobacteria;o__Alteromonadales;f__Alteromonadaceae;g__Alteromonas
2e69b4b8c8c5e5230d457c617a80f4ab	-0.78	0.013	d__Bacteria;p__Firmicutes;c__Bacilli;o__Izemoplasmatales;f__Izemoplasmataceae;g__Izimaplasma;s__uncultured Izimaplasma
8c20b9b4a39902fcc1ae0a4e34ee0a4e	-0.80	0.010	d__Bacteria;p__Bacteroidota;c__Bacteroidia;o__Flavobacteriales;f__Cryomorphaceae;g__Phaeocystidibacter

***Archaea*:**

ASV	Pearson Correlation	P-value	taxonomy
915aa73cc4f1ba83cef2cb31f5ea1062	0.97	0.002	d__*Archaea*;p__*Halobacterota*;c__*Halobacteria*;o__*Halobacterales*;f__*Halomicrobiaceae*;g__*Halapricum*
ca018f9f87f1b2613964b7d79af0a2df	0.96	0.002	d__*Archaea*;p__*Halobacterota*;c__*Halobacteria*;o__*Halobacterales*;f__*Haloferacaceae*;g__*Haloplanus*;s__uncultured *Haloplanus*
a49a8bd91ebd61f0794acf3da2568aec	0.96	0.003	d__*Archaea*;p__*Halobacterota*;c__*Halobacteria*;o__*Halobacterales*;f__*Haloferacaceae*;g__*Haloplanus*;s__uncultured *Haloplanus*
76df539101391133a38d72abdcbb0c23	0.95	0.004	d__*Archaea*;p__*Halobacterota*;c__*Halobacteria*;o__*Halobacterales*;f__*Haloferacaceae*;g__*Halobellus*;s__uncultured *Halobellus*
d049749b5278d20888a6dbcaec51f835	0.93	0.007	d__*Archaea*;p__*Halobacterota*;c__*Halobacteria*;o__*Halobacterales*;f__*Haloferacaceae*;g__*Halobellus*
49d8cc73ead043049db3c342ae42a699	0.93	0.008	d__*Archaea*;p__*Halobacterota*;c__*Halobacteria*;o__*Halobacterales*;f__*Haloferacaceae*;g__*Halorubrum*
262e1c5b89a9bc1a77cffc791875dd1e	0.92	0.010	d__*Archaea*;p__*Halobacterota*;c__*Halobacteria*;o__*Halobacterales*;f__*Haloferacaceae*;g__*Haloplanus*;s__uncultured *Haloplanus*
703bfc369b52686be14ed09c24df7d55	0.91	0.011	d__*Archaea*;p__*Halobacterota*;c__*Halobacteria*;o__*Halobacterales*;f__*Haloferacaceae*;g__*Haloplanus*;s__*Haloplanus_sp.*
6aa1a4ae0da8f76dca7a29e8f9c70857	0.91	0.013	d__*Archaea*;p__*Halobacterota*;c__*Halobacteria*;o__*Halobacterales*;f__*Haloferacaceae*;g__*Haloplanus*
17b7647f7de8c21fec9b6c2aa14e6c77	0.90	0.015	d__*Archaea*;p__*Halobacterota*;c__*Halobacteria*;o__*Halobacterales*;f__*Haloferacaceae*;g__*Haloplanus*;s__uncultured *Haloplanus*
7ebdc6af4f353477b542f6983f5f9e5c	0.90	0.015	d__*Archaea*;p__*Halobacterota*;c__*Halobacteria*;o__*Halobacterales*;f__*Halomicrobiaceae*;g__*Halapricum*;s__uncultured *Halapricum*
d5c885c41d53a91bf7a1321b7cab09c7	0.89	0.016	d__*Archaea*;p__*Halobacterota*;c__*Halobacteria*;o__*Halobacterales*;f__*Haloferacaceae*;g__*Halobellus*;s__uncultured *Halobellus*
4a350830e87730f00ddf6bd30fbb2799	0.89	0.016	d__*Archaea*;p__*Halobacterota*;c__*Halobacteria*;o__*Halobacterales*;f__*Halomicrobiaceae*;g__*Natronomonas*;s__uncultured *Natronomonas*
0c6add50ff5e77902aabde9d4654e17b	0.89	0.017	d__*Archaea*;p__*Halobacterota*;c__*Halobacteria*;o__*Halobacterales*;f__*Haloferacaceae*;g__*Haloquadratum*
5c17f84513f90bc63667b88ec91dd999	0.88	0.020	d__*Archaea*;p__*Halobacterota*;c__*Halobacteria*;o__*Halobacterales*;f__*Halobacterales*;g__uncultured *Halobacterales*;s__uncultured *Halobacterales*
6d9d52cabf2578b1abcd85e3d713307d	0.88	0.020	d__*Archaea*;p__*Halobacterota*;c__*Halobacteria*;o__*Halobacterales*;f__*Haloferacaceae*;g__*Haloplanus*
783146ad4e5f9ec18b63385b193c65ee	0.87	0.024	d__*Archaea*;p__*Halobacterota*;c__*Halobacteria*;o__*Halobacterales*;f__*Halobacterales*;g__uncultured *Halobacterales*
12174d7f1ced3b549478e28255017a14	0.87	0.024	d__*Archaea*;p__*Halobacterota*;c__*Halobacteria*;o__*Halobacterales*;f__*Haloferacaceae*;g__*Haloplanus*;s__uncultured *Haloplanus*
ecc9b026cd2680fa18587576ee4c34f1	0.87	0.024	d__*Archaea*;p__*Halobacterota*;c__*Halobacteria*;o__*Halobacterales*;f__*Halococcaceae*;g__*Halococcus*;s__uncultured *Halococcus*
fd3c0dc24923533308a5e34c5a80690c	0.87	0.025	d__*Archaea*;p__*Halobacterota*;c__*Halobacteria*;o__*Halobacterales*;f__*Halobacterales*;g__uncultured *Halobacterales*
16c452e96f771a7a9fa218a2d487521f	0.87	0.026	d__*Archaea*;p__*Nanohaloarchaeota*;c__*Nanosalinia*;o__*Nanosalinales*;f__*Nanosalinaceae*;g__*Nanosalinaceae*;s__uncultured *Nanosalinaceae*
3dabb665c3a7f8a544b5dc4d7118b4ce	0.85	0.034	d__*Archaea*;p__*Halobacterota*;c__*Halobacteria*;o__*Halobacterales*;f__*Haloferacaceae*;g__*Halobellus*;s__uncultured *Halobellus*
73f9bdf2fa500fced5dd37dc7217ed18	0.85	0.034	d__*Archaea*;p__*Halobacterota*;c__*Halobacteria*;o__*Halobacterales*;f__*Haloferacaceae*;g__*Haloplanus*;s__uncultured *Haloplanus*
8c6fee6a317e640e294ed4d99daf276d	0.84	0.035	d__*Archaea*;p__*Halobacterota*;c__*Halobacteria*;o__*Halobacterales*
1be7b1c781cac2b29578d55cc251dce8	0.84	0.035	d__*Archaea*;p__*Halobacterota*;c__*Halobacteria*;o__*Halobacterales*;f__*Halomicrobiaceae*;g__*Halapricum*;s__uncultured *Halapricum*
6bc26dd7410b686f291f8100eb57c5f8	0.84	0.035	d__*Archaea*;p__*Halobacterota*;c__*Halobacteria*;o__*Halobacterales*;f__*Haloferacaceae*;g__*Haloquadratum*
d1035bc334303ce23721677da8f722f6	0.83	0.039	d__*Archaea*;p__*Halobacterota*;c__*Halobacteria*;o__*Halobacterales*;f__*Haloferacaceae*;g__*Halorubrum*;s__uncultured *Halorubrum*

0ee966c0882ec6969d0d5d3fb94003b3	0.83	0.039	d__*Archaea*;p__*Halobacterota*;c__*Halobacteria*;o__*Halobacterales*;f__*Haloferacaceae*;g__*Haloquadratum*
f816cc791191aa39bf671e1d70e32164	0.83	0.041	d__*Archaea*;p__*Halobacterota*;c__*Halobacteria*;o__*Halobacterales*;f__*Halomicrobiaceae*;g__uncultured *Halomicrobiaceae*;s__*Halomicroarcula_sp.*
f290beb60494ab94f9ce57ca7512676d	0.82	0.046	d__*Archaea*;p__*Halobacterota*;c__*Halobacteria*;o__*Halobacterales*;f__*Halomicrobiaceae*;g__*Halapricum*;s__uncultured *Halapricum*
d5668ef5271e6d386f749812b4c0b2fc	0.82	0.048	d__*Archaea*;p__*Halobacterota*;c__*Halobacteria*;o__*Halobacterales*;f__*Halomicrobiaceae*;g__*Halapricum*;s__uncultured *Halapricum*
477af6b666b5e847cd81f9afd6be6c1b	0.81	0.050	d__*Archaea*;p__*Halobacterota*;c__*Halobacteria*;o__*Halobacterales*;f__*Halomicrobiaceae*;g__*Halomicroarcula*;s__*Halomicroarcula_sp.*
a98eda4127fec71276cd970ec62264f9	-0.82	0.048	d__*Archaea*;p__*Nanoarchaeota*;c__*Nanoarchaeia*;o__*Woesearchaeales*;f__*Woesearchaeales*;g__*Woesearchaeales*;s__uncultured *Woesearchaeales*
08332dbb947a584cd23c6da4de658f62	-0.82	0.048	d__*Archaea*;p__*Nanoarchaeota*;c__*Nanoarchaeia*;o__*Woesearchaeales*;f__*Woesearchaeales*;g__*Woesearchaeales*;s__uncultured *Woesearchaeales*
ae51480ebc650b47691282f0269a3411	-0.82	0.043	d__*Archaea*;p__*Nanoarchaeota*;c__*Nanoarchaeia*;o__*Woesearchaeales*;f__*Woesearchaeales*;g__*Woesearchaeales*;s__uncultured *Woesearchaeales*
586d364926de38c880d1163850dd9a81	-0.84	0.038	d__*Archaea*;p__*Nanoarchaeota*;c__*Nanoarchaeia*;o__*Woesearchaeales*;f__*SCGC_AAA286-E23*;g__*SCGC_AAA286-E23*;s__uncultured *SCGC_AAA286-E23*
c2984c7c61260e1930788bf6e31b3788	-0.84	0.036	d__*Archaea*;p__*Nanoarchaeota*;c__*Nanoarchaeia*;o__*Woesearchaeales*;f__*SCGC_AAA286-E23*;g__*SCGC_AAA286-E23*
35a745929095d7e1b41b30e4e8cc3160	-0.85	0.030	d__*Archaea*;p__*Nanoarchaeota*;c__*Nanoarchaeia*;o__*Woesearchaeales*;f__*Woesearchaeales*;g__*Woesearchaeales*;s__uncultured *Woesearchaeales*
c24193857c7d70e01c9f12e163768429	-0.86	0.030	d__*Archaea*;p__*Nanoarchaeota*;c__*Nanoarchaeia*;o__*Woesearchaeales*;f__*Woesearchaeales*;g__*Woesearchaeales*;s__uncultured *Woesearchaeales*
45f3c2b14b687d59cb0cfddeb6c25dbe	-0.86	0.029	d__*Archaea*;p__*Nanoarchaeota*;c__*Nanoarchaeia*;o__*Woesearchaeales*;f__*Woesearchaeales*;g__*Woesearchaeales*;s__uncultured *Woesearchaeales*
5a830c437b8f3d8a9bb64de8affc1a57	-0.86	0.029	d__*Archaea*;p__*Nanoarchaeota*;c__*Nanoarchaeia*;o__*Woesearchaeales*;f__*SCGC_AAA286-E23*;g__*SCGC_AAA286-E23*
eb50e33ff310a4bdc5fdc80f8ddae09e	-0.86	0.028	d__*Archaea*;p__*Nanoarchaeota*;c__*Nanoarchaeia*;o__*Woesearchaeales*;f__*SCGC_AAA286-E23*;g__*SCGC_AAA286-E23*
63d5522ff64f6b3c4d4bb647f844c845	-0.86	0.028	d__*Archaea*;p__*Nanoarchaeota*;c__*Nanoarchaeia*;o__*Woesearchaeales*;f__*Woesearchaeales*;g__*Woesearchaeales*;s__uncultured *Woesearchaeales*
f006c8e87e1cb5ed303f4f604f1e051b	-0.86	0.027	d__*Archaea*;p__*Nanoarchaeota*;c__*Nanoarchaeia*;o__*Woesearchaeales*;f__*Woesearchaeales*;g__*Woesearchaeales*;s__uncultured *Woesearchaeales*
5de53397fd9b23517077d90786daa91e	-0.86	0.026	d__*Archaea*;p__*Nanoarchaeota*;c__*Nanoarchaeia*;o__*Woesearchaeales*;f__*Woesearchaeales*;g__*Woesearchaeales*;s__uncultured *Woesearchaeales*
b5e3b86102d272a518768e5947dd887d	-0.87	0.026	d__*Archaea*;p__*Nanoarchaeota*;c__*Nanoarchaeia*;o__*Woesearchaeales*;f__*Woesearchaeales*;g__*Woesearchaeales*;s__uncultured *Woesearchaeales*
ca25dfcc580d5ae810f07bb4a0fab561	-0.87	0.024	d__*Archaea*;p__*Nanoarchaeota*;c__*Nanoarchaeia*;o__*Woesearchaeales*;f__*SCGC_AAA286-E23*;g__*SCGC_AAA286-E23*;s__uncultured *SCGC_AAA286-E23*
f78424c30233f2b7760e4ea79c59f2ec	-0.87	0.023	d__*Archaea*;p__*Halobacterota*;c__*Halobacteria*;o__*Halobacterales*;f__*Haloferacaceae*;g__uncultured *Haloferacaceae*;s__uncultured *Haloferacaceae*
c1b719c6514a7431e5381fcfceef56cb	-0.87	0.023	d__*Archaea*;p__*Nanoarchaeota*;c__*Nanoarchaeia*;o__*Woesearchaeales*;f__*Woesearchaeales*;g__*Woesearchaeales*;s__uncultured *Woesearchaeales*
76dcf6baead4e9cbd880e256b547f6d1	-0.88	0.021	d__*Archaea*;p__*Nanoarchaeota*;c__*Nanoarchaeia*;o__*Woesearchaeales*;f__*GW2011*;g__*AR15*;s__uncultured *AR15*
387e4d114e2484968674765833607afa	-0.88	0.021	d__*Archaea*;p__*Nanoarchaeota*;c__*Nanoarchaeia*;o__*Woesearchaeales*;f__*Woesearchaeales*;g__*Woesearchaeales*;s__uncultured *Woesearchaeales*
7a992d24b6fe6764300ee609db1a14a0	-0.88	0.020	d__*Archaea*;p__*Nanoarchaeota*;c__*Nanoarchaeia*;o__*Woesearchaeales*;f__*Woesearchaeales*;g__*Woesearchaeales*
32a01c7ee8c009c4e5014a52103ef45f	-0.88	0.020	d__*Archaea*;p__*Nanoarchaeota*;c__*Nanoarchaeia*;o__*Woesearchaeales*;f__*Woesearchaeales*;g__*Woesearchaeales*;s__uncultured *Woesearchaeales*
c94f6c522b9bcb2a9165b8b6462118c0	-0.89	0.018	d__*Archaea*;p__*Nanoarchaeota*;c__*Nanoarchaeia*;o__*Woesearchaeales*;f__*Woesearchaeales*;g__*Woesearchaeales*;s__uncultured *Woesearchaeales*
132cc77567d2d5dca75da0d2ca7ed2c1	-0.89	0.018	*d__Archaea;p__Aenigmarchaeota;c__Deep_Sea_Euryarchaeotic_Group(DSEG);o__Deep_Sea_Euryarchaeotic_Group(DSEG);f__Deep_Sea_ Euryarchaeotic_Group(DSEG);g__Deep_Sea_Euryarchaeotic_Group(DSEG);s__uncultured Deep_Sea_Euryarchaeotic_Group(DSEG)*

35a52acdec8f4e30ac7a5a8feccaaacf	-0.89	0.017	d__Archaea;p__Nanoarchaeota;c__Nanoarchaeia;o__Woesearchaeales;f__Woesearchaeales;g__Woesearchaeales
68a4981c5d14f3506f29b7ba4c21982f	-0.89	0.017	d__Archaea;p__Iainarchaeota;c__Iainarchaeia;o__Iainarchaeales;f__Iainarchaeales;g__Candidatus_Iainarchaeum;s__metagenome
d6019e724d7b2c7d3b38b40e3feeaa8e	-0.90	0.015	d__Archaea;p__Halobacterota;c__Halobacteria;o__Halobacterales;f__Halomicrobiaceae;g__Natronomonas;s__uncultured Natronomonas
9d21d400740fe36476bac8600e4c40eb	-0.90	0.015	d__Archaea;p__Nanoarchaeota;c__Nanoarchaeia;o__Woesearchaeales;f__Woesearchaeales;g__Woesearchaeales;s__uncultured Woesearchaeales
b64d79c9eb23ec53164ff0644c23c070	-0.90	0.015	d__Archaea;p__Nanoarchaeota;c__Nanoarchaeia;o__Woesearchaeales;f__Woesearchaeales;g__Woesearchaeales;s__uncultured Woesearchaeales
cf1f41d9e9acd422b3b7ef006024d907	-0.90	0.013	d__Archaea;p__Nanoarchaeota;c__Nanoarchaeia;o__Woesearchaeales;f__Woesearchaeales;g__Woesearchaeales;s__uncultured Woesearchaeales
bbf4959fc31c8226839b8ae97721f961	-0.91	0.013	d__Archaea;p__Nanoarchaeota;c__Nanoarchaeia;o__Woesearchaeales;f__Woesearchaeales;g__Woesearchaeales;s__uncultured Woesearchaeales
a7e3012a77db9e26760d6dbc8374d1aa	-0.91	0.012	d__Archaea;p__Nanoarchaeota;c__Nanoarchaeia;o__Woesearchaeales;f__Woesearchaeales;g__Woesearchaeales;s__uncultured Woesearchaeales
b4707fb5c3fde18a710f6699af1dbdf4	-0.91	0.012	d__Archaea;p__Halobacterota;c__Halobacteria;o__Halobacterales;f__Haloferacaceae;g__Haloterrigena;s__Haloterrigena_hispanica
82e84c861f76ea436ab9b6170397faba	-0.91	0.012	d__Archaea;p__Nanoarchaeota;c__Nanoarchaeia;o__Woesearchaeales;f__Woesearchaeales;g__Woesearchaeales;s__uncultured Woesearchaeales
5b3ee5898068da70c958aeb81dd10a12	-0.91	0.012	d__Archaea;p__Nanoarchaeota;c__Nanoarchaeia;o__Woesearchaeales;f__Woesearchaeales;g__Woesearchaeales;s__uncultured Woesearchaeales
4a6e347dc8cf80daa333e2332bd8e47f	-0.92	0.010	d__Archaea;p__Halobacterota;c__Halobacteria;o__Halobacterales;f__Halomicrobiaceae;g__Natronomonas;s__uncultured Natronomonas
8828cb9fe570a42d894c3ae297ebd706	-0.93	0.006	d__Archaea;p__Halobacterota;c__Halobacteria;o__Halobacterales;f__Haloferacaceae;g__uncultured Haloferacaceae;s__uncultured Haloferacaceae

Table S5. Relative abundances of bacterial and archaeal genera in the ponds of the MdS saltern obtained by metabarcoding analysis. Ponds are indicated by conventional names, with salinity reported as salt percentage (w/v). Groups with relative abundances < 1.0 % were joined as ‘Others’. ASVs which could not be resolved at the genus level were reported with the notation g_unclassified followed by the name of the closest known parental rank.

***Bacteria*:**

**Phylum**	**Genus**	**Alma Dannata (4.9%)**	**Polmone (5.2%)**	**Zero (8.4%)**	**Fine (13.1%)**	**Paradiso (14.5%)**	**Inizio (24.1%)**	**Armellina (30.6%)**	**Cappella (34.6%)**	**Imperatrice (36.0%)**
	Candidatus_Aquiluna	19.33%	15.09%	9.27%	22.74%	25.98%	1.72%			
*Actinobacteriota**	*ML602J-51*	1.87%	1.48%							
	Pontimonas				2.10%	2.23%				
	g_uncultured *Chitinophagales*						6.49%	15.90%	12.21%	11.41%
	Brumimicrobium			4.05%						
	Owenweeksia		1.36%		1.70%	1.56%				
	g_uncultured *Cryomorphaceae*	1.09%	2.23%							
	NS3a_marine_group	6.69%	2.08%							
	NS5_marine_group	3.59%								
*Bacteroidota***	*Psychroflexus*			3.56%	18.17%	14.09%	4.21%			
	ML602M-17			1.52%	1.62%					
	Balneola		1.88%	1.34%						
	Gracilimonas			4.12%	3.20%	2.01%				
	g_uncultured *Balneolaceae*		2.09%	1.82%	3.14%	2.41%				
	Salinibacter						52.85%	79.00%	86.86%	88.18%
	Peredibacter		1.43%							
*Bdellovibrionota****	*Candidatus_Izimaplasma*	4.45%	4.46%	1.40%						
Firmicutes	*Izemoplasmatales*		3.86%							
	Cohaesibacter	1.42%								
	Donghicola		3.69%							
	Marivita			1.35%						
	Roseovarius			4.86%	3.23%	3.55%				
	WDS1C4				3.00%	3.09%				
	AEGEAN-169_marine_group	2.05%	1.29%							
	Clade_III	14.10%	3.41%	1.03%						
	Alteromonas	1.04%	1.14%							
	Idiomarina			2.43%						
	Pseudoalteromonas	7.65%		7.71%						
Proteobacteria	g_uncultured *Alcaligenaceae*		14.70%							
	Halioglobus	1.65%								
	Luminiphilus		1.97%							
	g_uncultured Unknown_Family				2.73%	3.12%				
	Spiribacter					2.24%	12.47%			
	Methylophaga				2.25%	1.11%				
	Cobetia	2.26%								
	Halomonas			3.87%	5.34%	7.35%				
	Litoricola	11.39%	8.55%	1.04%		1.23%				
	Salinivibrio			8.30%	5.22%	1.62%				

Verrucomicrobiota	g_uncultured *Simkaniaceae*		1.58%							
	Lentimonas			1.55%						
	Others	6.47%	13.41%	29.43%	6.15%	6.34%	3.60%	2.88%	0.93%	0.41%
	Unclassified	14.96%	14.32%	11.35%	19.43%	22.09%	18.66%	2.23%		

*~~)~~ also known as *Actinobacteria*; **~~)~~ also known as *Bacteroidetes*; ***~~)~~ also known as *Tenericutes*.

***Archaea*:**

**Phylum**	**Genus**	**Fine (13.1%)**	**Paradiso (14.5%)**	**Inizio (24.1%)**	**Armellina (30.6%)**	**Cappella (34.6%)**	**Imperatrice (36.0%)**
*Halobacterota**	g_uncultured *Halobacterales*			1.44%	1.64%	4.92%	9.97%
	g_uncultured *Haloferacaceae*	41.57%	17.41%	3.88%			
	Haloquadratum	2.18%	31.66%	40.77%	37.37%	53.40%	63.52%
	Haloplanus				1.61%	1.54%	1.31%
	Halobellus		1.09%	2.21%	4.79%	3.61%	2.77%
	Halorubrum	3.17%	5.96%	10.36%	14.47%	8.82%	5.43%
	Halonotius		6.24%	11.20%	10.99%	9.57%	3.95%
	Halomarina			1.47%	1.76%		
	Natronomonas	49.23%	25.09%	18.83%	18.78%	8.26%	3.22%
	Halomicroarcula			1.36%	1.06%	1.25%	
	Halapricum			1.43%	1.68%	4.01%	5.05%
Nanoarchaeota	*Woesearchaeales*	1.44%	1.63%				
	SCGC_AAA286-E23	1.60%					
Nanohaloarchaeota	*Candidatus_ Nanosalinarum*		3.93%				1.05%
	Nanosalinaceae		5.02%	4.08%	2.19%		1.17%
	Others	0.82%	1.96%	2.95%	2.27%	4.61%	2.56%
	Unclassified				1.38%		

*~~)~~ also known as *Euryarchaeota*.

Table S6. Relative abundances of bacterial and archaeal genera in the ponds of the MdS saltern obtained by combination of metabarcoding and CARD-FISH data. Ponds are indicated by conventional names, with salinity reported as salt percentage (w/v). Groups with relative abundances < 1.0 % were joined as ‘Others’. ASVs which could not be resolved at the genus level were reported with the notation g_unclassified followed by the name of the closest known parental rank.

**Domain**	**Phylum**	**Genus**	**Fine (13.1%)**	**Paradiso (14.5%)**	**Inizio (24.1%)**	**Armellina (30.6%)**	**Cappella (34.6%)**	**Imperatrice (36.0%)**
Bacteria	*Actinobacteriota**	*Candidatus_Aquiluna*	14.61%	19.24%				
		Pontimonas	1.35%	1.65%				
	*Bacteroidota***	g_uncultured *Chitinophagales*			2.62%	9.02%	6.85%	3.75%
		Owenweeksia	1.09%	1.15%				
		Psychroflexus	11.67%	10.44%	1.70%			
		ML602M-17	1.04%					
		Gracilimonas	2.05%	1.49%				
		g_uncultured *Balneolaceae*	2.02%	1.79%				
		Salinibacter			21.34%	44.82%	48.76%	28.98%
	Proteobacteria	*Roseovarius*	2.08%	2.63%				
		WDS1C4	1.92%	2.29%				
		g_uncultured Unknown_Family	1.75%	2.31%				
		Spiribacter		1.66%	5.03%			
		Methylophaga	1.44%					
		Halomonas	3.43%	5.44%				
		Salinivibrio	3.35%	1.20%				
		Unclassified_B	12.48%	16.36%	7.54%	1.27%		
		Others_B	3.95%	6.42%	2.15%	1.63%	0.52%	0.13%
Archaea	*Halobacterota****	g_uncultured *Halobacterales*					2.16%	6.69%
		g_uncultured *Haloferacaceae*	14.87%	4.51%	2.32%			
		Haloquadratum		8.21%	24.31%	16.17%	23.43%	42.64%
		Halobellus			1.32%	2.07%	1.58%	1.86%
		Halorubrum	1.13%	1.54%	6.18%	6.26%	3.87%	3.64%
		Halonotius		1.62%	6.68%	4.76%	4.20%	2.65%
		Natronomonas	17.61%	6.51%	11.23%	8.12%	3.62%	2.16%
		Halapricum					1.76%	3.39%
	Nanohaloarchaeota	*Candidatus_Nanosalinarum*		1.02%				
		Nanosalinaceae		1.30%	2.43%			
		Others_A	2.16%	1.22%	5.16%	5.88%	3.25%	4.09%

*) also known as *Actinobacteria*; **) also known as *Bacteroidetes*; ***) also known as *Euryarchaeota*.

Figure S1

A **B**

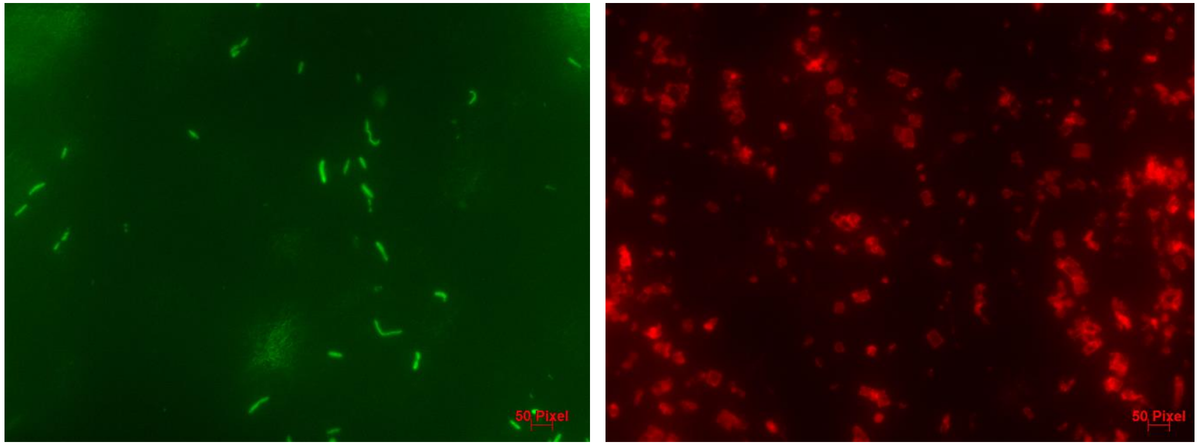

Figure S1. Detection of bacterial and archaeal cells by CARD-FISH. Prokaryotic cells (A: *Bacteria*; B: *Archaea*) in the waters of the Imperatrice pond (36.0% of salinity) as visualized by CARD-FISH analysis. Among *Bacteria*, rod-shaped cells should mostly correspond to *Salinibacter* cells according to the high abundance of the genus (about 88%); numerous square-shaped *Haloquadratum* cells are distinguishable among archaeal cells.

Figure S2

A

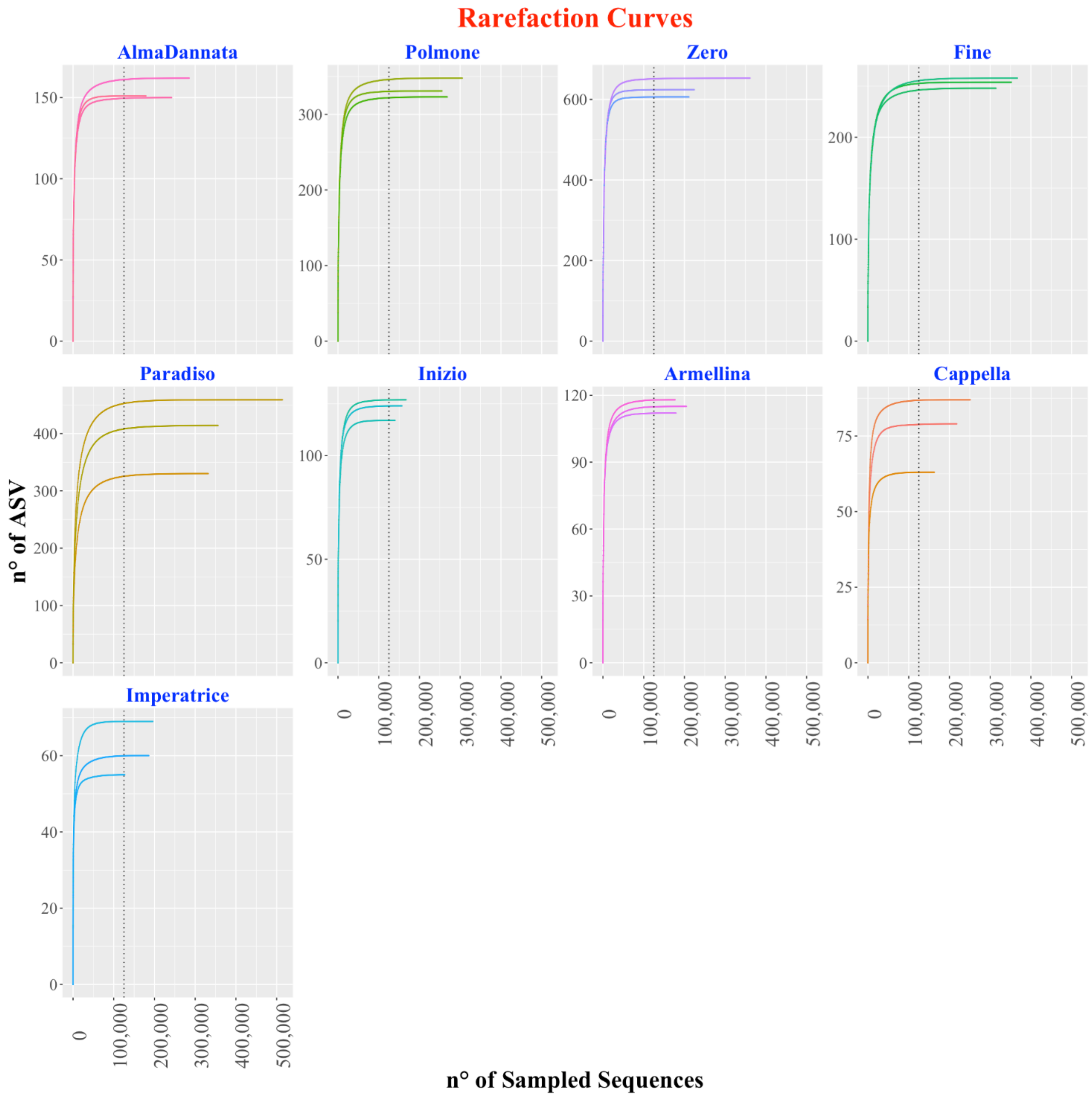

Rarefaction Curves
AlmaDannata
Polmone
Zero
Fine
Paradiso
Inizio
Armellina
Cappella
Imperatrice
n° of ASV
n° of Sampled Sequences
0
100,000
200,000
300,000
400,000
500,000

B

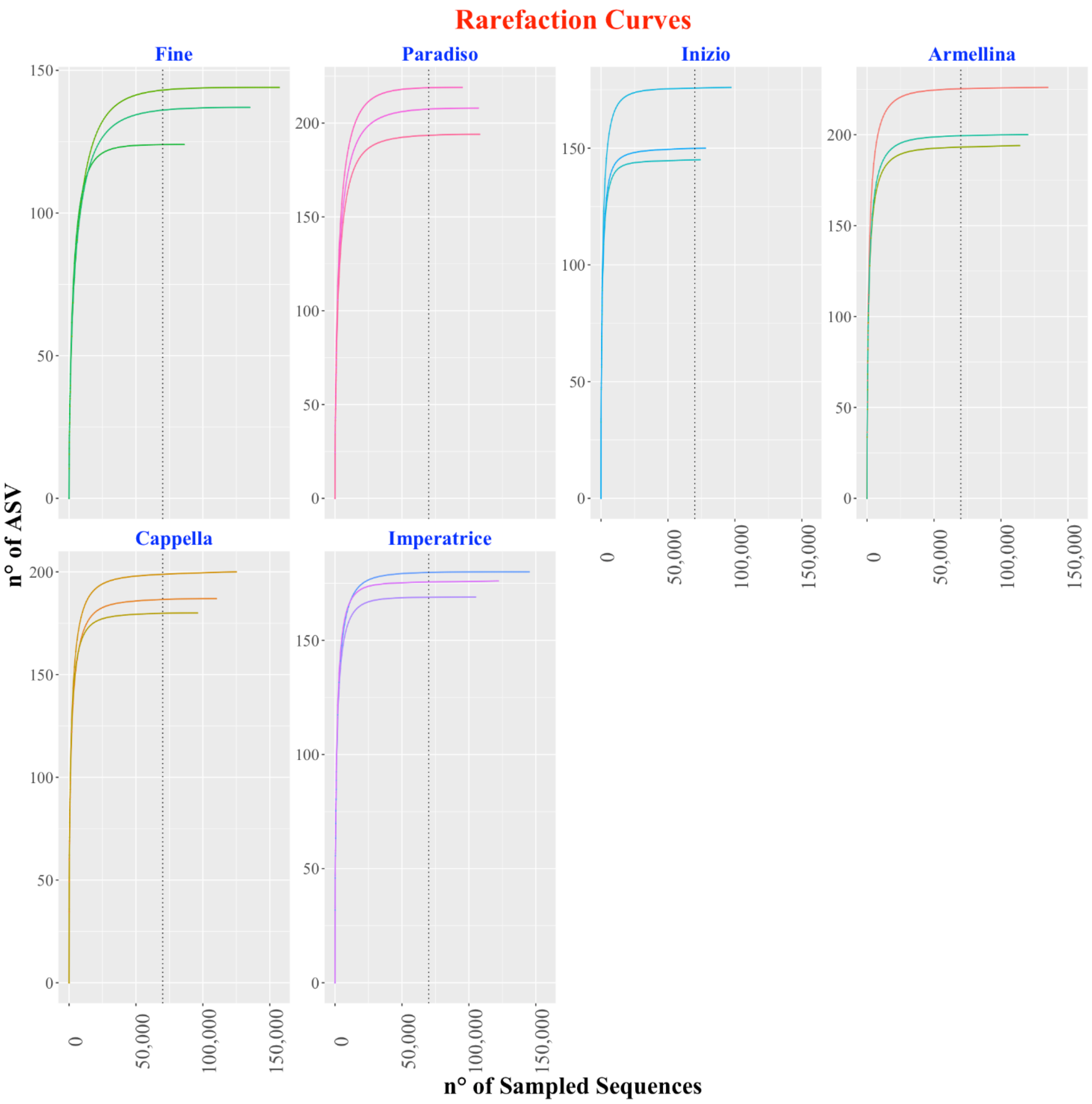

Figure S2. Rarefaction curves of 16S rRNA ASVs for the assayed ponds of the MdS saltern. A) bacterial ASVs; B) archaeal ASVs.

Figure S3

A

B

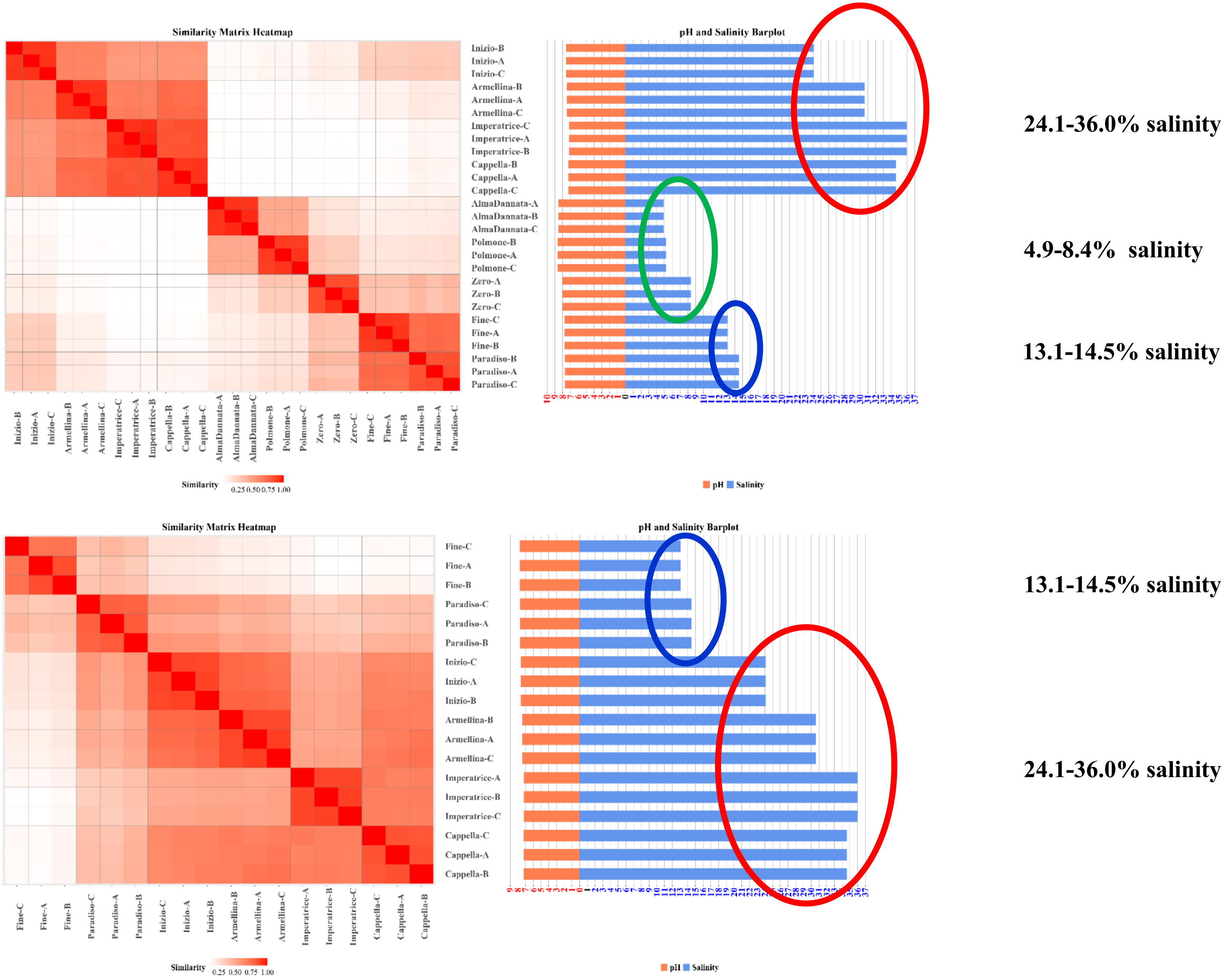
Similarity Matrix Heatmap
pH and Salinity Barplot
Inizio-B
Inizio-A
Inizio-C
Armellina-B
Armellina-A
Armellina-C
Imperatrice-C
Imperatrice-A
Imperatrice-B
Cappella-B
Cappella-A
Cappella-C
AlmaDannata-A
AlmaDannata-B
AlmaDannata-C
Polmone-B
Polmone-A
Polmone-C
Zero-A
Zero-B
Zero-C
Fine-C
Fine-A
Fine-B
Paradiso-B
Paradiso-A
Paradiso-C
Similarity
0.25 0.50 0.75 1.00
pH
Salinity
24.1-36.0% salinity
4.9-8.4% salinity
13.1-14.5% salinity

Figure S3. Heat maps of microbial amplicons similarity matrix of MdS ponds. The heat maps (A: *Bacteria*; B: *Archaea*) were obtained by inferring the Bray-Curtis distances on ASV counts and clustering the samples using the UPGMA algorithm. The letters A, B and C accompanying the names of the ponds refer to the values of the experimental replicas. On the right of the heat maps, corresponding salinity and pH values are shown (coloured cycles define pond salinity ranges).
